# βA1-crystallin regulates glucose metabolism and mitochondrial function in mouse retinal astrocytes by modulating PTP1B activity

**DOI:** 10.1038/s42003-021-01763-5

**Published:** 2021-02-24

**Authors:** Sayan Ghosh, Haitao Liu, Meysam Yazdankhah, Nadezda Stepicheva, Peng Shang, Tanuja Vaidya, Stacey Hose, Urvi Gupta, Michael Joseph Calderon, Ming-Wen Hu, Archana Padmanabhan Nair, Joseph Weiss, Christopher S. Fitting, Imran A. Bhutto, Santosh Gopi Krishna Gadde, Naveen Kumar Naik, Chaitra Jaydev, Gerard A. Lutty, James T. Handa, Ashwath Jayagopal, Jiang Qian, José-Alain Sahel, Dhivyaa Rajasundaram, Yuri Sergeev, J. Samuel Zigler, Swaminathan Sethu, Simon Watkins, Arkasubhra Ghosh, Debasish Sinha

**Affiliations:** 1 Department of Ophthalmology, University of Pittsburgh School of Medicine, Pittsburgh, PA USA; 2 GROW Research Laboratory, Narayana Nethralaya Foundation, Bengaluru, India; 3 Department of Cell Biology and Center for Biologic Imaging, University of Pittsburgh School of Medicine, Pittsburgh, PA USA; 4 Wilmer Eye Institute, The Johns Hopkins University School of Medicine, Baltimore, MD USA; 5 Kodiak Sciences, Palo Alto, CA USA; 6 Institut de la Vision, INSERM, CNRS, Sorbonne Université, Paris, France; 7 Department of Pediatrics, Children’s Hospital of Pittsburgh, University of Pittsburgh School of Medicine, Pittsburgh, PA USA; 8 National Eye Institute, National Institutes of Health, Bethesda, MD USA

**Keywords:** Molecular medicine, Cell signalling

## Abstract

βA3/A1-crystallin, a lens protein that is also expressed in astrocytes, is produced as βA3 and βA1-crystallin isoforms by leaky ribosomal scanning. In a previous human proteome high-throughput array, we found that βA3/A1-crystallin interacts with protein tyrosine phosphatase 1B (PTP1B), a key regulator of glucose metabolism. This prompted us to explore possible roles of βA3/A1-crystallin in metabolism of retinal astrocytes. We found that βA1-crystallin acts as an uncompetitive inhibitor of PTP1B, but βA3-crystallin does not. Loss of βA1-crystallin in astrocytes triggers metabolic abnormalities and inflammation. In CRISPR/cas9 gene-edited βA1-knockdown (KD) mice, but not in βA3-knockout (KO) mice, the streptozotocin (STZ)-induced diabetic retinopathy (DR)-like phenotype is exacerbated. Here, we have identified βA1-crystallin as a regulator of PTP1B; loss of this regulation may be a new mechanism by which astrocytes contribute to DR. Interestingly, proliferative diabetic retinopathy (PDR) patients showed reduced βA1-crystallin and higher levels of PTP1B in the vitreous humor.

## Abstract (web summary)

Ghosh et al. show that a lens protein βA1-crystallin inhibits PTP1B activity, regulating the metabolism of retinal astrocytes. They find that knockdown of βA1-crystallin exacerbates the streptozotocin-induced diabetic retinopathy-like phenotype in mice, suggesting the possibility that the interaction between βA1-PTP1B may be targeted for diabetic retinopathy.

## Introduction

βA3/A1-crystallin is unusual amongst eukaryotic proteins, in that its gene, *Cryba1*, produces two proteins via leaky ribosomal scanning^1,2^. The two isoforms, βA3 and βA1, are identical in primary sequence, except that βA3-crystallin has an additional 17 amino acid residues at its amino terminus^2^. At present, the cellular function of βA3/A1-crystallin is uncertain and whether the two isoforms could have distinct functions has not been explored. In the lens, which recruited *Cryba1* later in evolution, the two isoforms function interchangeably as structural elements. However, leaky ribosomal scanning is sometimes used to control the expression of regulatory genes^2,3^, and thus it is possible that *Cryba1* is an important regulatory gene in cells where it does not appear to be a structural protein, such as astrocytes. In a previous human proteome high-throughput array (CDI Laboratories, Inc.), we found that βA3/A1-crystallin interacts with PTP1B^4^, an enzyme that links metabolism and inflammation in diabetes^5^. PTP1B is ubiquitously expressed and is a known negative regulator of the leptin and insulin signaling pathways^6^. Increased PTP1B phosphatase activity has been implicated in several disease processes, including diabetic retinopathy (DR)^7,8^. In multifactorial diseases, such as DR, pathological remodeling of affected tissue is often of pivotal importance in the disease etiology^9^. One important gap in our understanding of the different stages of DR progression is the potential role of the retinal glial population.

Astrocytes are one of the two types of glial cells found in mammalian retinas^10^. Unlike Müller cells, which span the entire thickness of the retina and are present in all mammals, astrocytes are mainly confined to the retina’s inner surface and are closely associated with retinal blood vessels^10^. Astrocytes are known to be involved in various neurodegenerative diseases such as Alzheimer’s and Parkinson’s disease^11,12^. In addition, astrocytes are known to play a key role in diabetes-associated conditions such as diabetic neuropathy and DR^13,14^. During development in rodents, astrocytes migrate into the retina through the optic nerve head as a mixture of precursor cells and immature perinatal astrocytes and then spread across the nerve fiber layer towards peripheral margins of the retina, where they contribute critically to retinal angiogenesis and the formation of the brain-retinal-barrier^15^.

We have previously shown that abnormal functioning of astrocytes due to lack of functional βA3/A1-crystallin leads to alterations in the developing retinal vasculature^10,16^. However, the underlying signaling mechanism(s) by which βA3- or βA1-crystallin could modulate astrocyte function in the pathological retinal remodeling is not known. To determine if the βA1 and βA3 isoforms have distinct functions, we generated βA3-crystallin knockout (βA3 KO) and βA1-crystallin knockdown (βA1 KD) mice by CRISPR/cas9 gene editing. PTP1B activity is increased in normal and high glucose (HG) treated βA1 KD astrocytes compared to WT cells in vitro, while βA3 KO astrocytes show no noticeable change in PTP1B activity compared to WT cells.

Further we show that knockdown of βA1-crystallin in mice triggers substantial alterations in glucose metabolism and mitochondrial function in the astrocytes via the dysregulation of the PTP1B/signal transducer and activator of transcription 3 (STAT3) signaling axis, which is known to be critical for maintaining cellular metabolic regulation^17,18^. These alterations in glucose metabolism in the astrocytes cause inflammation and might be associated with the onset of a DR-like pathology seen in the retinas of βA1 KD mice, but not observed in βA3 KO mice. Our data suggest that abnormality in the association between βA1-crystallin and PTP1B might exacerbate DR pathology both in our mouse model and in human PDR patients. Taken together these results suggest that βA1-crystallin is important for normal astrocyte function and maintenance of retinal homeostasis.

## Results

### βA1-crystallin binds to PTP1B and regulates its activity

**Fig. 1:**
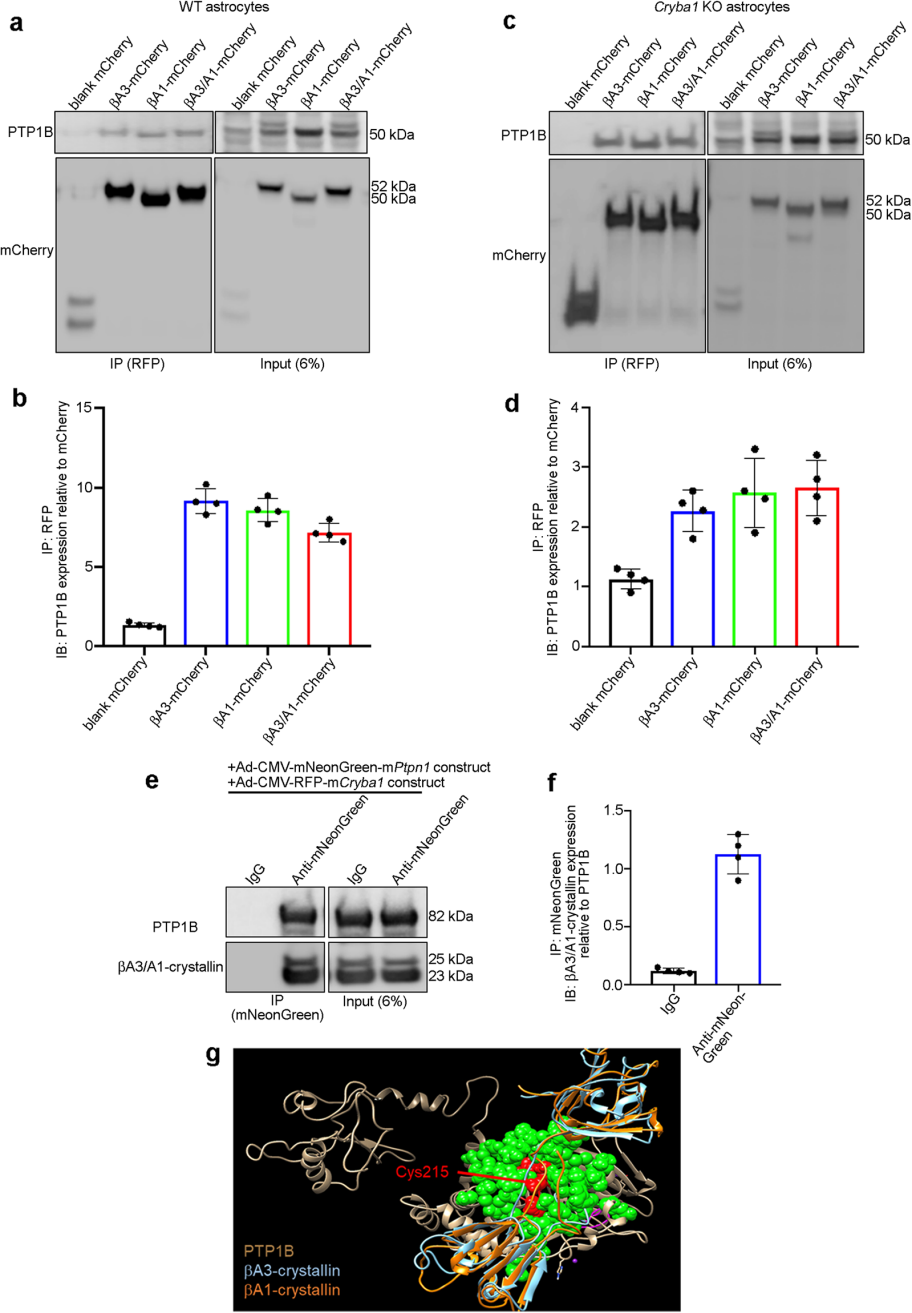
βA1-crystallin is a binding partner of PTP1B. Representative western blot and densitometry graph from co-immunoprecipitation studies in mouse WT (**a**, **b**) or *Cryba1* KO (**c**, **d**) astrocytes transfected with blank-mCherry, βA3-crystallin-mCherry (βA3-mCherry), βA1-crystallin-mCherry (βA1-mCherry), and βA3/A1-crystallin-mCherry (βA3/A1-mCherry) show interaction of PTP1B with both βA3- and βA1-crystallin; *n* = 4. **e**, **f** Co-immunoprecipitation assay showing βA3/A1-crystallin levels in the Co-IP eluent by western blot analysis and densitometry, indicating binding to PTP1B, upon pull down with mNeonGreen antibody-bound magnetic beads from lysates of astrocytes overexpressing PTP1B (Ad-CMV-mNeonGreen-m*Ptpn1*) and *Cryba1* (Ad-CMV-RFP-m*Cryba1*). Pull down with mouse IgG showed no binding for βA3/A1-crystallin; *n* = 4. **g** Ribbon diagram obtained by molecular modeling showing superimposed βA1-crystallin (orange), βA3-crystallin (blue), and PTP1B (gray). Neither isoform is able to bind to the pocket of PTP1B active site (Cys215, Red). The βA1, but not the βA3 isoform (due to a steric bump of terminal extension) interacts with an allosteric binding site (green) on the surface of PTP1B.

We have previously shown that βA3/A1-crystallin regulates retinal astrocyte function^10,19^, but whether the βA3 and βA1 isoforms have distinct roles in astrocytes has not been studied. In a human proteome high-throughput array we found that βA3/A1-crystallin interacts with PTP1B^4^, an enzyme that links glucose metabolism and inflammation in diabetes^5^. To evaluate possible differential binding status of PTP1B to the βA1 and βA3 isoforms, we transfected wild type (WT) and *Cryba1* knockout (KO) astrocytes with blank mCherry, βA3-mCherry, βA1-mCherry, or βA3/A1-mCherry constructs. We used *Cryba1* KO astrocytes for the co-immunoprecipitation (Co-IP) assay to determine which βA3/A1-crystallin isoforms are binding partners of PTP1B. The pull down was performed with anti-red fluorescent protein (RFP) beads from the lysates of these transfected cells and the levels of PTP1B and mCherry (co-immunoprecipitation control) were assessed by immunoblotting, showing that PTP1B binds to both βA3 and βA1 isoforms in WT and *Cryba1* KO astrocytes (Fig. 1a–d). We also performed a reverse Co-IP assay, in which PTP1B and βA3/A1-crystallin were overexpressed in WT mouse astrocytes using Ad-CMV-mNeonGreen-m*Ptpn1* and Ad-CMV-RFP-m*Cryba1* constructs. Anti-mNeonGreen antibody-bound magnetic beads and mouse IgG beads were prepared and were used to pull down PTP1B from the astrocyte lysates. The βA3/A1-crystallin level was evaluated by western blot in the co-immunoprecipitated eluent (Fig. 1e, f). The findings confirm binding between PTP1B and βA3/A1-crystallin. In addition, molecular modeling supports an interaction between these PTP1B and βA3/A1-crystallin isoforms (Fig. 1g). The βA1 isoform in open conformation (orange) could interact with an allosteric binding site (green) at the surface of PTP1B, yet is unable to bind to the pocket of the PTP1B active site (red, Cys215). Moreover, the βA3 isoform (blue) is unable to interact at the allosteric site due to a steric bump of the terminal extension^20^ and PTP1B (gray), indicating decreased βA3 binding.

**Fig. 2:**
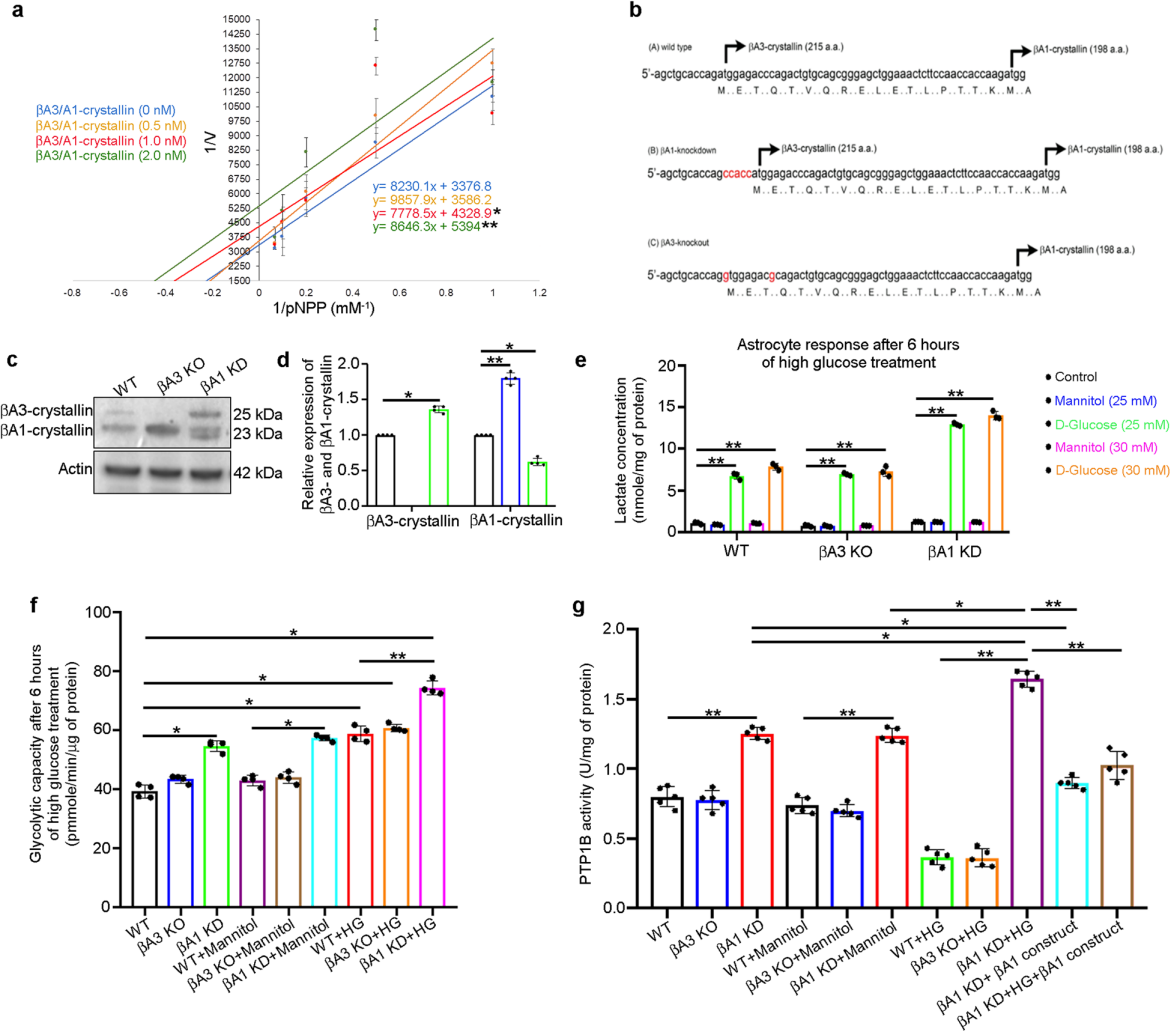
βA1-crystallin regulates PTP1B activity. **a** Lineweaver-Burk plot showing increasing doses (0, 0.5, 1, and 2 nM) of βA3/A1-crystallin decreases both *V*_max_ and *K*_M_ of PTP1B activity for different concentrations of p-Nitrophenyl Phosphate (pNPP). *n* = 3. *P < 0.05. **P < 0.05. **b** The N-terminal sequence of *Cryba1*; βA1-crystallin KD mice were generated by knocking in 5 base pairs (CCACC, red) before the first start codon to strengthen the Kozak consensus sequence. For generating βA3-crystallin KO, the first start codon was removed by a single nucleotide mutation in the mouse *Cryba1* gene (A > G, red). Another silent mutation (C > G; red) was also introduced to prevent the binding and re-cutting of the sequence by gRNA after homology-directed repair. **c** Representative western blot and **d** graph showing densitometry analysis for the expression level of βA3/A1-crystallin in astrocyte lysates from WT (black bar), βA3 KO (blue bar), and βA1 KD (green bar) mice, respectively, showing complete loss of βA3-crystallin in the βA3 KO cells and a notable decrease in βA1-crystallin expression in the βA1 KD cells, relative to WT astrocytes. In βA3 KO astrocytes, there is an increase in expression of βA1-crystallin; *n* = 4. *P < 0.05, **P < 0.01. **e** Increased levels of lactate in mouse WT, βA3 KO and βA1 KD astrocytes treated with HG (25 or 30 mM for 6 h) relative to untreated cells. Lactate levels in βA1 KD astrocytes were higher in all experimental conditions, compared to WT and βA3 KO cells; *n* = 3. *P < 0.05, **P < 0.01. **f** Elevated glycolytic flux is evident from increased glycolytic capacity in WT, βA3 KO and βA1 KD astrocytes treated with high glucose (HG; 30 mM for 6 h), relative to untreated cells (cultured in 5 mM d-glucose containing medium). Glycolytic capacity in βA1 KD astrocytes was drastically higher compared to WT and βA3 KO cells; *n* = 4. *P < 0.05, **P < 0.01. **g** Cultured βA1 astrocytes either untreated or exposed to mannitol (30 mM for 6 h) have elevated PTP1B activity compared to WT and βA3 KO cells, which increases further with HG (30 mM for 6 h). The elevation in PTP1B activity was rescued by βA1-crystallin overexpression in untreated or HG-exposed βA1 KD cells; *n* = 5. *P < 0.05, **P < 0.01.

The pull-down assay and molecular docking clearly suggest that βA3/A1-crystallin binds to PTP1B and that the βA1 isoform in particular could possibly bind to an allosteric site close to the active site (Cys215, which is essential for its activity) of the enzyme. This finding led us to hypothesize that βA3/A1-crystallin could regulate the activity of PTP1B. By enzyme kinetic analysis (Lineweaver-Burk Plot) using para-nitrophenyl phosphate (p-NPP) as the substrate for PTP1B, we found that βA3/A1-crystallin is an uncompetitive inhibitor of human PTP1B enzyme activity as indicated by decreased *V*_max_ and *K*_M_ with increasing doses of recombinant human βA3/A1-crystallin (Fig. 2a). To further delineate whether the βA3 or βA1 isoforms have distinct roles in regulating PTP1B activity and glucose metabolism in astrocytes, we generated βA3 KO and βA1 KD mice by CRISPR/cas9 genome editing as described in Fig. 2b; the level of βA1-crystallin is notably upregulated in βA3 KO astrocytes (Fig. 2c, d), suggesting a possible compensation mechanism in these cells.

To evaluate the specific capacity of βA3- or βA1-crystallin to regulate PTP1B activity and glucose metabolism in retinal astrocytes, we first assessed the response to HG in these cells relative to the osmolarity control (mannitol). We performed a time and dose-dependent estimation of the glycolytic end product, lactate^21^, in WT, βA3 KO, and βA1 KD mouse astrocytes. We observed that retinal astrocytes from all three genotypes, when exposed to 25 or 30 mM HG for 0.5, 1, 1.5, 2, 3, 6, 12, or 24 h, showed elevated lactate levels after 6 h of HG exposure (Supplementary Fig. 1a–c and Fig. 2e), compared to untreated cells (cultured in 5 mM d-Glucose containing medium). Lactate levels declined at later time points (Supplementary Fig. 1a–c). In addition, no noteworthy differences were observed in the lactate levels between the 25 and 30 mM doses of glucose in astrocytes from any of the three mouse genotypes (Supplementary Fig. 1a–c and Fig. 2e). Hence, in all subsequent cell culture experiments involving HG, 30 mM glucose was used to treat retinal astrocytes from different genotypes for a duration of 6 h, while mannitol was used as the osmolarity control.

Further, we assessed the metabolic flux in these cells by using the glycolytic stress assay kit (Seahorse XF platform; Agilent, USA). The glycolytic capacity, which measures the maximum extracellular acidification rate (ECAR) reached by a cell following the addition of oligomycin (thereby effectively shutting down oxidative phosphorylation and driving the cell to use glycolysis to its maximum capacity), was substantially increased in mouse astrocytes from all three genotypes, when exposed to HG (30 mM) for 6 h, relative to untreated cells (Fig. 2f). Glycolytic capacity was notably higher in untreated or HG-treated βA1 KD astrocytes compared to WT and βA3 KO cells (Fig. 2f). Interestingly, the lactate levels (Supplementary Fig. 1a–c and Fig. 2e) in HG-treated WT and βA3 KO astrocytes were similar to those in untreated cells at 24 h, but remained higher in βA1 KD astrocytes even after 24 h (Supplementary Fig. 1a–c). Taken together, these results indicate that in comparison to WT and βA3 KO astrocytes, cells from βA1 KD mice are unable to metabolize HG efficiently and produce higher levels of lactate, which in turn has been associated with astrocyte activation^22^.

Further, to assess PTP1B activity, WT, βA3 KO and βA1 KD astrocytes were either left untreated or were treated with 30 mM of mannitol or HG, for 6 h. βA1 KD astrocytes, untreated or treated (with mannitol or HG), showed elevated PTP1B activity relative to WT cells that received the same treatments (Fig. 2g). Surprisingly, HG-exposed WT and βA3 KO astrocytes showed decreased levels of PTP1B activity compared to HG-exposed βA1 KD cells (Fig. 2g). Overexpression of the βA1-mCherry construct in untreated or HG-exposed βA1 KD cells rescued the abnormally high PTP1B activity in these cells (Fig. 2g). These results indicate that the presence of βA1-crystallin is critical in regulating PTP1B activity under basal and hyperglycaemic stress conditions, probably owing to the ability of βA1-crystallin to bind to the allosteric site of the PTP1B enzyme (Fig. 1g).

### βA1-crystallin regulates glucose metaboli**s**m in retinal astrocytes

**Fig. 3:**
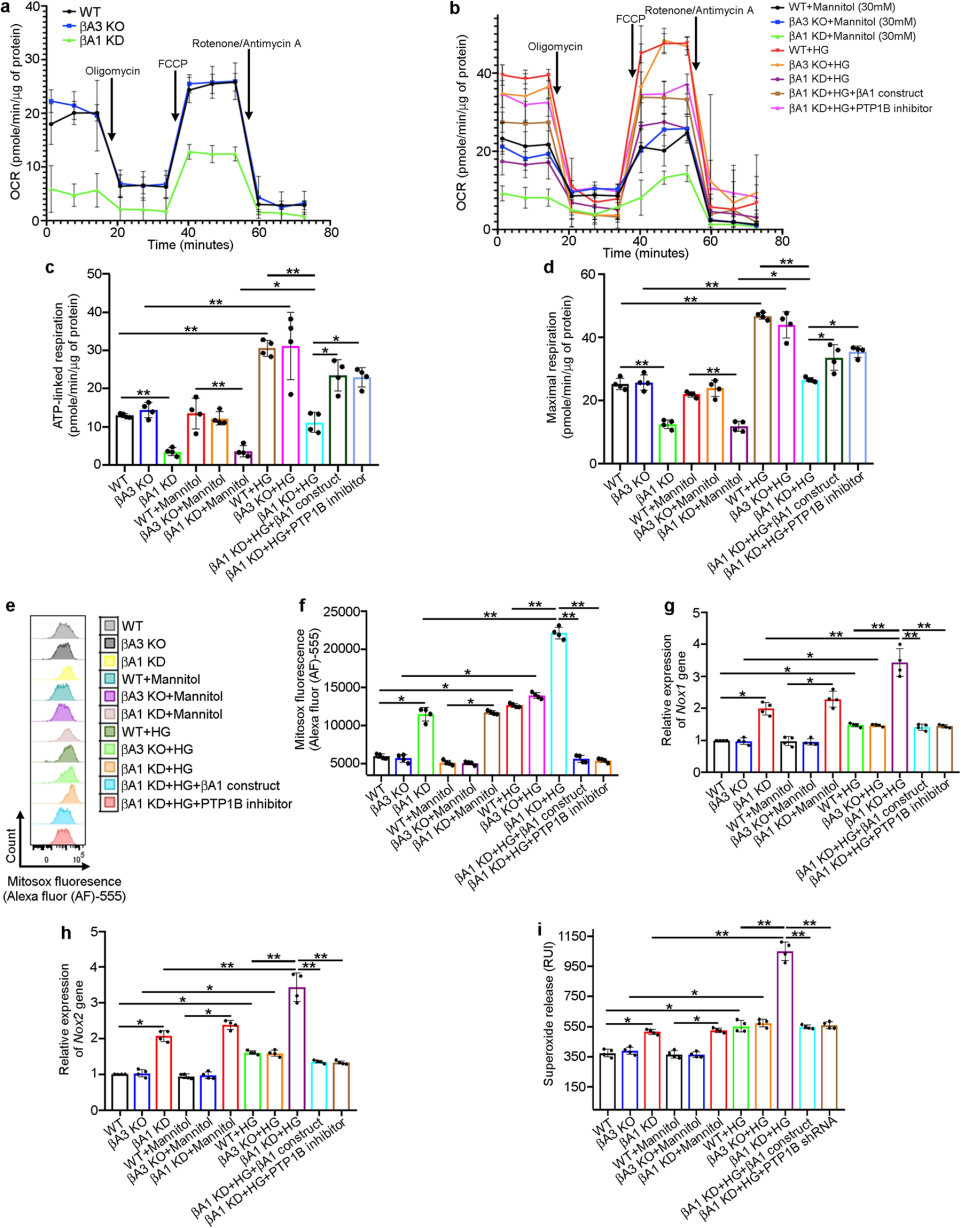
βA1-crystallin regulates glucose metabolism in retinal astrocytes. **a**, **b** Plots from seahorse analysis using the mitostress assay showing time-dependent changes in metabolic flux upon treatment with mitochondrial respiration blockers oligomycin, Carbonyl cyanide-p-trifluoromethoxyphenylhydrazone (FCCP), Rotenone/Antimycin A at particular time points (indicated by arrows). *Y*-axis denotes oxygen consumption rate (OCR; pmole/min/μg) and *X*-axis represents time (minutes) for **a** untreated (cultured in 5 mM d-Glucose) WT, βA3 KO, and βA1 KD mouse astrocytes or **b** astrocytes exposed to either mannitol (30 mM for 6 h) or high glucose (HG; 30 mM for 6 h) respectively, or βA1 astrocytes transfected with mCherry-βA1 overexpression construct for 48 h or treated with 10 μM of PTP1B inhibitor (MSI-1436) for 1 h prior to HG (30 mM) exposure for 6 h; *n* = 4. Reduced mitochondrial function is shown by decreased **c** ATP-linked respiration and **d** maximal respiration, in untreated or mannitol (30 mM for 6 h) and HG (30 mM for 6 h)-exposed βA1 KD astrocytes, compared to WT cells. WT and βA3 KO astrocytes treated with HG showed an increase in both **c** ATP-linked respiration and **d** maximal respiration, compared to untreated cells. In βA1 KD astrocytes transfected with βA1-mCherry overexpression construct for 48 h or treated with 10 μM of PTP1B inhibitor (MSI-1436) for 1 h prior to HG (30 mM) exposure for 6 h, the levels of **c** ATP-linked respiration and **d** maximal respiration were partially rescued; *n* = 4. *P < 0.05, **P < 0.01. **e**, **f** Flow cytometry histograms and graph for MitoSox fluorescence (Alexa fluor (AF)-555), **g** *Nox1* gene expression, **h** *Nox2* gene expression and **i** superoxide release in βA1 KD astrocytes either untreated or exposed to mannitol (30 mM for 6 h) showed increased levels of **e**, **f** mROS, **g** *Nox1* along with **h** *Nox2* gene expression and **i** superoxide release, that increased further with HG (30 mM for 6 h) exposure, relative to WT cells (**e**–**h**). βA1-crystallin overexpression (using βA1-mCherry construct) or PTP1B inhibition in βA1 KD astrocytes followed by treatment with HG, reduced the elevated levels of **e**, **f** mROS, **g** *Nox1*, and **h** *Nox2* gene expression and **i** superoxide release in βA1 KD cells. *n* = 4. *P < 0.05, **P < 0.01.

Elevated PTP1B activity is known to disrupt mitochondrial respiration and glucose metabolism^23,24^. Thus, our observations that PTP1B activity is modulated by βA1-crystallin encouraged us to investigate mitochondrial function in retinal astrocytes upon hyperglycemic stress. We therefore measured mitochondrial flux in retinal astrocytes from WT, βA3 KO, and βA1 KD mice using the Mito-Stress assay kit (Seahorse XF platform; Agilent, USA) to evaluate the oxygen consumption rate (OCR), a measure of mitochondrial function or oxidative phosphorylation (OxPhos) when mitochondrial activity is sequentially blocked. We found a substantial decrease in OCR levels along with ATP-linked respiration (measure of ATP production) and maximal respiration in βA1 KD astrocytes with or without 6 h of HG treatment relative to WT cells (Fig. 3a–d). However, HG exposure markedly increased the mitochondrial function (OCR, ATP-linked respiration, and maximal respiration levels) in HG-treated WT and βA3 KO cells compared to untreated cells (Fig. 3a–d), suggesting that βA1-crystallin is essential for maintaining mitochondrial function in astrocytes under hyperglycemic stress. Moreover, to further pinpoint the importance of the βA1 isoform in mitochondrial function and glucose metabolism in retinal astrocytes, we pre-transfected βA1 KD astrocytes with the βA1-mCherry construct for 48 h or treated them with an inhibitor of PTP1B activity (MSI-1436) at a dose of 10 μM for 1 h^24^, followed by HG treatment for 6 h. Our results revealed that βA1-crystallin overexpression or PTP1B inhibition in HG-exposed βA1 KD cells rescued the OCR, ATP-linked respiration and maximal respiration levels to near WT values (Fig. 3a–d). Mannitol-treated cells did not show any noteworthy change in mitochondrial function relative to untreated astrocytes (Fig. 3a–d).

Alterations in mitochondrial function and decreased ATP production by OxPhos leads to generation of excessive superoxide anion (O_2_^•−^)/mitochondrial reactive oxygen species (mROS)^25^. The generation of O_2_^•−^ within the mitochondrial matrix depends critically on protonmotive force (Δ*p*)^25^. When mitochondria are not making ATP and consequently have a high Δ*p*, the level of O_2_^•−^ production increases^25^. As the βA1 KD astrocytes showed diminished mitochondrial function, we evaluated the mROS production in WT, βA3 KO and βA1 KD astrocytes and found that HG exposure to astrocytes from all three genotypes increased the intracellular levels of mROS (Fig. 3e, f). However, the increase in mROS in βA1 KD astrocytes that were left untreated or were treated with 30 mM of HG or mannitol for 6 h, was considerably higher relative to WT and βA3 KO astrocytes (Fig. 3e, f). Moreover, βA3 KO cells did not show any noticeable change in mROS production when compared to WT astrocytes (Fig. 3e, f). Further, overexpression of the βA1-mCherry construct or treatment with the PTP1B inhibitor in βA1 KD cells followed by HG treatment for 6 h reduced the levels of mROS back to normal (Fig. 3e, f).

In general, mitochondria act as a redox sink and function by limiting cellular NADPH oxidase (NOX) activity^26^. However, with an increase in mROS, NADPH oxidases are activated^26^. This crosstalk between mitochondria and NADPH oxidases represents a feed-forward cycle of ROS production causing oxidative stress^26^. Astrocytes, in particular, have abundant levels of Nox1 and Nox2 isoforms^27^. Nox isoforms are localized in the plasma membrane and release excess superoxide radical (O_2_^•−^) from the cell because their active site faces the extracellular space^28^. As mROS increases upon HG treatment in these retinal astrocytes, we evaluated the levels of *Nox1* and *Nox2* gene expression and superoxide release in the WT, βA3 KO, and βA1 KD astrocytes and observed increased expression of *Nox1* and *Nox2* genes and of superoxide release in HG-exposed astrocytes from all three genotypes (Fig. 3g–i). While βA3 KO astrocytes did not show any noticeable change relative to WT cells (Fig. 3g–i), the increase in *Nox1* and *Nox2* gene expression along with superoxide release in βA1 KD astrocytes that were left untreated or were treated with 30 mM glucose or mannitol for 6 h, was higher than that of WT or βA3 KO cells exposed to the same treatment (Fig. 3g–i). However, βA1-crystallin overexpression or treatment with PTP1B inhibitor prior to HG exposure in βA1 KD astrocytes rescued the *Nox1* and *Nox2* gene expression as well as the superoxide levels (Fig. 3g–i). These results highlight the importance of βA1-crystallin in regulating mitochondrial function, energy production and oxidative stress in retinal astrocytes during HG stress.

### Abnormal STAT3 signaling in βA1 KD astrocytes

**Fig. 4:**
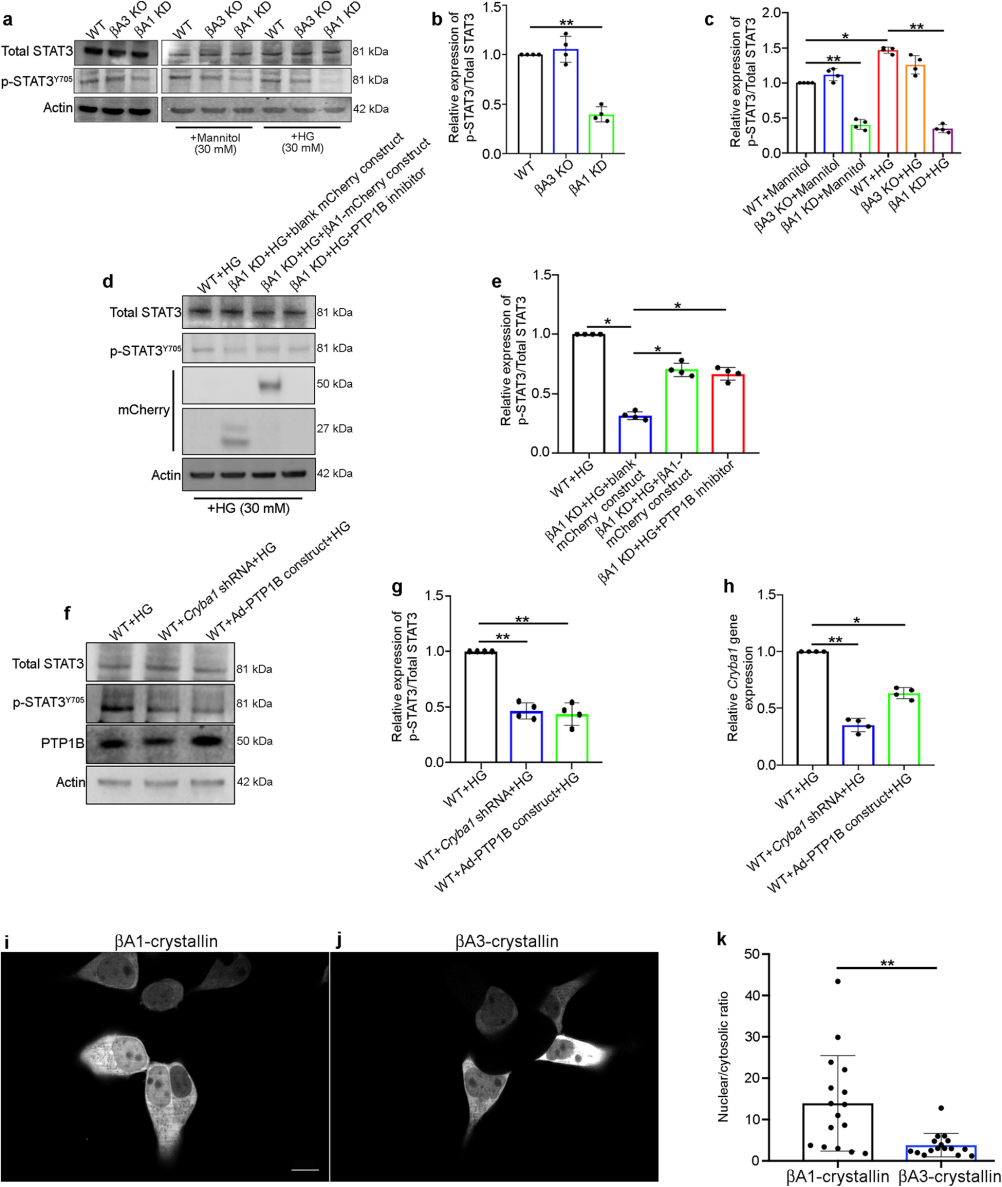
βA1-crystallin regulates STAT3 nuclear localization. **a** Representative western blot and **b**, **c** densitometry graphs showing decreased phosphorylation of STAT3 at tyrosine 705 (p-STAT3^Y705^) in βA1 KD astrocytes **b** untreated or **c** treated with 30 mM mannitol or high glucose (HG; 30 mM) for 6 h, relative to WT cells (**c**). βA3 KO cells did not show such changes (**a**–**c**); *n* = 4. **P < 0.01. **d**, **e** Overexpression of βA1-crystallin (using βA1-mCherry construct) in βA1 KD astrocytes or treatment with 10 μM PTP1B inhibitor (MSI-1436) for 1 h, prior to HG (30 mM) exposure for 6 h rescued the levels of p-STAT3^Y705^, as compared to βA1 KD cells transfected with blank-mCherry construct. βA1-crystallin overexpression was confirmed by mCherry western blot. **f** Western blot and **g** densitometry graph showing decrease in p-STAT3^Y705^ expression in WT cells infected with Adenovirus-PTP1B overexpression construct or *Cryba1* shRNA for 48 h, followed by HG treatment. **h** *Cryba1* knockdown was confirmed by qPCR, which showed about 70% downregulation compared to control. *n* = 4. *P < 0.05, **P < 0.01. **i**, **j** Representative images from live-cell confocal microscopy of human iPSC-derived astrocytes after overexpression of **i** βA1-mCherry or **j** βA3-mCherry constructs and **k** quantitative assessment of nuclear translocation, showing nuclear localization of βA1-crystallin in these cells (**i**, **k**), whereas βA3-crystallin construct transfected cells showed less nuclear localization (**j**, **k**). Scale bar, 10  μm. **P < 0.01 (*n* = 16).

PTP1B acts upstream of the transcription factor STAT3^29^. Specifically, it is known that PTP1B dephosphorylates Jak2, thereby inhibiting STAT3 phosphorylation at tyrosine705 (Y705)^29^ that is critical for transcriptional regulation of genes involved in glucose metabolism^30,31^. In this context, we investigated the impact of the βA3 and βA1 isoforms on the STAT3 signaling pathway in retinal astrocytes and found that the levels of STAT3 phosphorylation (p-STAT3^Y705^) were appreciably decreased in βA1 KD astrocytes that were either left untreated or were treated with 30 mM of glucose (HG) for 6 h, as evident from the decrease in the p-STAT3^Y705^/total STAT3 ratio (Fig. 4a–c). In contrast, βA3 KO astrocytes did not show any noticeable change relative to WT cells (Fig. 4a–c). Mannitol-treated cells also did not show any substantial change in the levels of p-STAT3^Y705^ relative to untreated cells (Fig. 4a, c). The decreased STAT3 phosphorylation in HG-exposed βA1 KD cells was substantially rescued by either βA1-crystallin overexpression or PTP1B activity inhibition (Fig. 4d, e). In addition, the βA1-crystallin overexpression was confirmed in the cells by performing a western blot for mCherry, which showed a higher molecular weight band (50 kDa) for the βA1-mCherry construct and a lower molecular weight band (27 kDa) for the blank mCherry construct overexpression in the βA1 KD cells (Fig. 4d). To further show that PTP1B and βA3/A1-crystallin are important for STAT3 phosphorylation, we overexpressed PTP1B or knocked down *Cryba1* (by lentiviral shRNA particles) for 48 h in WT astrocytes and then exposed the cells to HG (30 mM) for 6 h. We observed reduced phosphorylation of STAT3 in the PTP1B overexpressed and *Cryba1* KD cells, relative to HG-treated WT cells (Fig. 4f, g). The downregulation of *Cryba1* in these cells upon infection with lentiviral shRNA was confirmed by qPCR (Fig. 4h). Interestingly, overexpression of PTP1B in WT astrocytes also resulted in decreased *Cryba1* expression (Fig. 4h), probably owing to the diminished nuclear activity (phosphorylation) of STAT3, which is known to bind to the *Cryba1* promoter in astrocytes and regulate its expression^32^. These results provide further evidence that PTP1B controls STAT3 phosphorylation in astrocytes, which in turn may be involved in regulating expression of βA3/A1-crystallin and other STAT3-dependent genes.

Moreover, we have previously shown that both STAT3 and βA3/A1-crystallin are co-regulated in the cytosol of astrocytes^32^. βA3/A1-crystallin is required for the phosphorylation of STAT3, which then dimerizes and translocates to the nucleus to form DNA-binding complexes, activating transcription of *Cryba1*^32^. This co-regulation between STAT3 and *Cryba1* in the astrocytes could potentiate the expression of genes required for maintaining cellular homeostasis. Further, βA3/A1-crystallin translocates to the nucleus in the astrocytes and this is thought be an important regulatory process in these cells^10,19^. However, the relative propensity for nuclear translocation of βA1- and βA3-crystallin isoforms is still unknown. To evaluate the ability of either of the crystallin isoforms to translocate into the nucleus, we overexpressed βA1 and βA3 polypeptides separately in a human astrocyte cell line and then performed live-cell imaging. Interestingly, quantitative assessment of nuclear translocation of the two isoforms showed that βA1-crystallin translocated to the nucleus (Fig. 4i, k and Supplementary Movie 1a), whereas βA3-crystallin was predominantly observed in the cytoplasm (Fig. 4j, k and Supplementary Movie 1b). This suggests that βA1-crystallin is of much greater importance than βA3-crystallin in modulating STAT3 nuclear translocation and thereby regulating the hyperglycemic stress response and cellular homeostasis in astrocytes.

**Fig. 5:**
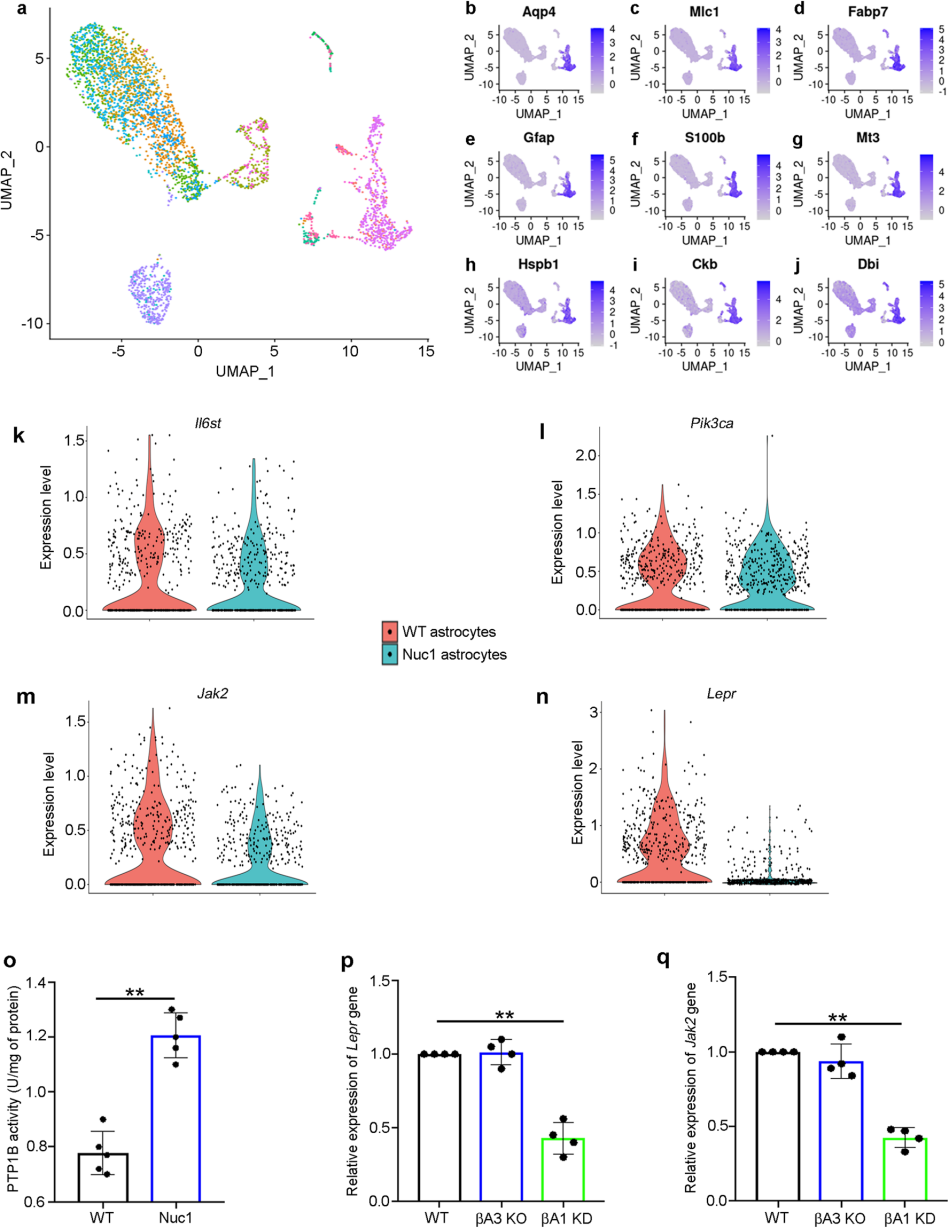
βA1-crystallin regulates STAT3-dependent signaling in retinal astrocytes. Integrated analysis of single-cell transcriptomic data across WT and Nuc1 genotype. **a** T-distributed stochastic neighbor embedding (t-SNE) plot of mixed cell culture samples harvested from the optic nerve head of WT and Nuc1 rats, integrated into a single dataset and clustered using Seurat. **b**–**j** Feature plots of gene markers used to identify and annotate astrocyte cell clusters. **k**–**n** Gene expression patterns of **k** *Il6st*, **l** *Pik3ca*, **m** *Jak2*, and **n** *Lepr* in WT and Nuc1 astrocytes displayed as violin plots. **o** Elevated PTP1B activity in cultured Nuc1 astrocytes, relative to WT. *n* = 4. *P* < 0.01. **p**, **q** qPCR analyses showing decreased expression of STAT3-dependent genes, **p** *Lepr* and **q** *Jak2* in cultured mouse βA1 KD astrocytes compared to WT cells. Astrocytes from mouse βA3 KO did not show any change in the expression of **p** *Lepr* and **q** *Jak2* genes, compared to WT cells. *n* = 4. *P* < 0.01.

Alterations in STAT3 nuclear localization are known to decrease downstream STAT3 target gene expression^32,33^. We previously showed that in Nuc1 rat (spontaneous insertion mutation in *Cryba1* gene) astrocytes, STAT3 signaling is abnormal^32^. To further reveal the changes in STAT3 signaling in retinal astrocytes, we have performed single-cell RNA sequencing (scRNAseq) from a mixed culture of cells from the optic nerve head of WT and Nuc1 rats. We performed differential expression analysis using Seurat and assessed the expression of differentially expressed genes in the STAT3 pathway specifically in the cell cluster which expresses all the essential markers for pan-astrocytes such as *Aqp4*, *Mlc1*, *Fabp7*, *Gfap*, *S100b*, *Mt3*, *Hspb1*, *Ckb*, and *Dbi* (Fig. 5a–j). Our results revealed that the upstream genes in the STAT3 signaling pathway such as *Il6st* (Interleukin 6 Signal Transducer)^34^ or *Pik3ca* (Phosphatidylinositol-4,5-Bisphosphate 3-Kinase Catalytic Subunit Alpha)^35^ did not show any noticeable change in expression between the WT and Nuc1 genotypes (Fig. 5k, l). In addition, the expression of known genes in the STAT3 pathway, such as, *Jak2* (Janus Kinase 2)^36^ and *Lepr* (leptin receptor)^37^, which are also known to be involved in regulating metabolism in cells^38,39^, were downregulated in the Nuc1 astrocytes (Fig. 5m, n), indicating that loss of functional βA3/A1-crystallin triggers abnormality in STAT3 signaling. It is likely that even though there is no upstream inhibitory signal for the JAK2/STAT3 signaling in Nuc1 astrocytes (Fig. 5k, l), the nuclear translocation of STAT3 in these cells is still abnormal because of the high PTP1B activity in these cells (Fig. 5o). Further, to evaluate the role of βA3 or βA1 isoforms on STAT3-dependent gene expression, we performed qPCR to quantify the expression of *Jak2* and *Lepr* genes in WT, βA3 KO and βA1 KD mouse astrocytes and found that both genes were downregulated in βA1 KD astrocytes while there was no noticeable change in βA3 KO cells compared to WT (Fig. 5p, q). This further substantiates the essential role of βA1-crystallin in regulating STAT3 signaling in retinal astrocytes.

### Activation of inflammation in βA1 KD astrocytes

**Fig. 6:**
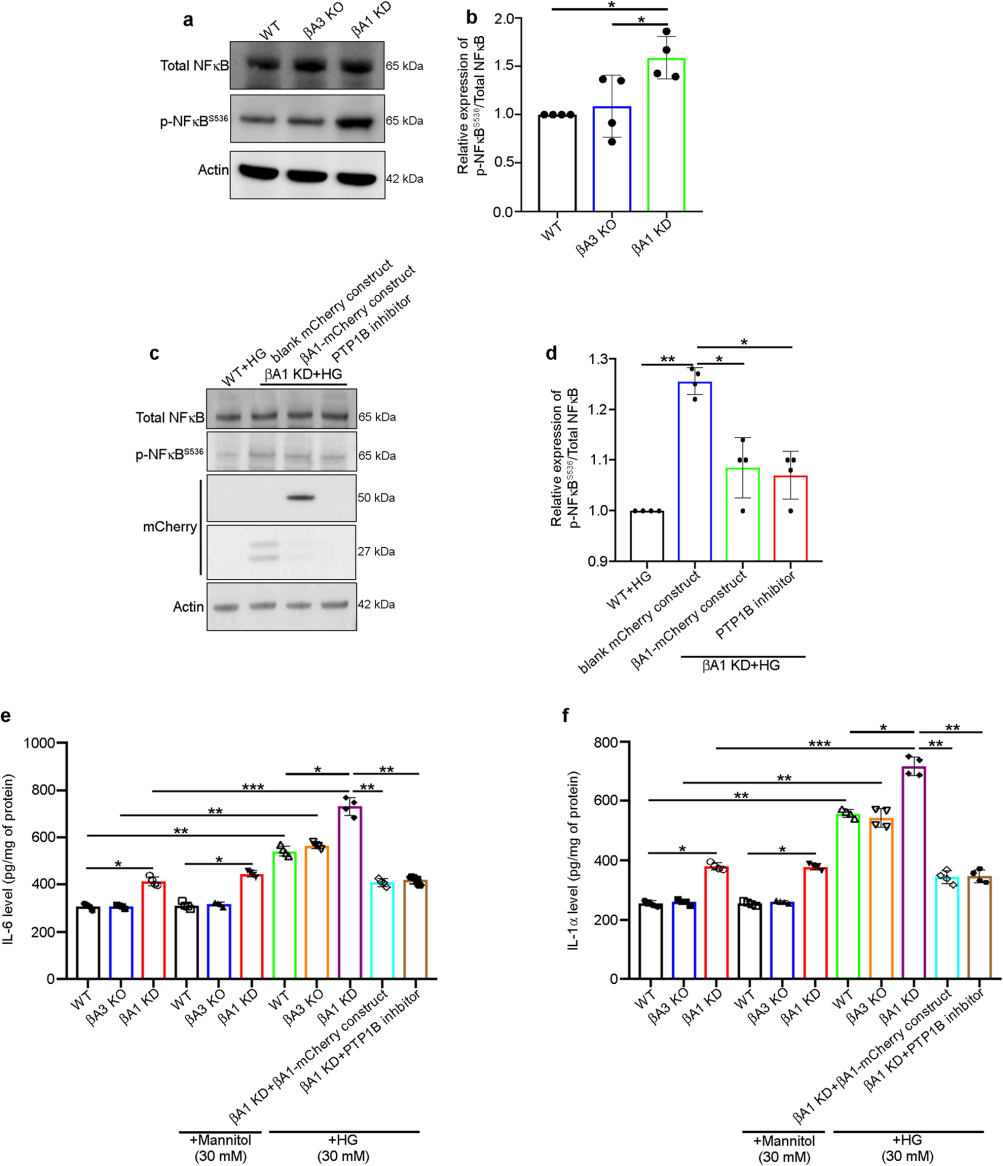
βA1-crystallin regulates inflammation in retinal astrocytes. **a** Representative western blot and **b** densitometry graph showing increased phosphorylation of NFκB at serine 536 (p-NFκB^S536^) in untreated (cultured in 5 mM d-Glucose containing medium) βA1 KD astrocytes, relative to WT cells. βA3 KO cells did not show such change in p-NFκB^S536^ levels. *n* = 4. *P < 0.05. **c**, **d** βA1 KD astrocytes transfected with βA1-mCherry construct for 48 h or treated with 10 μM of PTP1B inhibitor (MSI-1436) for 1 h, prior to HG (30 mM) exposure for 6 h rescued the levels of p-NFκB^S536^, compared to βA1 KD cells transfected with blank mCherry construct and then exposed to HG. βA1-crystallin overexpression was confirmed by mCherry western blot. *n* = 4. *P < 0.05, **P < 0.01. **e** IL-6 and **f** IL-1α levels showed marked increase in HG-exposed WT, βA3 KO, and βA1 KD astrocytes, compared to untreated cells. βA1 KD astrocytes showed increased levels of both cytokines relative to WT or βA3 KO cells. (**e**, **f**) Overexpression of βA1-crystallin (using βA1-mCherry construct) in βA1 KD astrocytes or treatment with 10 μM of PTP1B inhibitor (MSI-1436) for 1 h, prior to HG (30 mM) exposure for 6 h, rescued the levels of IL-6 and IL-1α. *n* = 4. *P < 0.05, **P < 0.01.

Increased mROS production triggers inflammation^40^. NFκB is known to regulate inflammatory processes in astrocytes and upregulates the pro-inflammatory cytokines like, IL-6 and IL-1α which have been shown to play a critical role in DR^41,42^. We observed that astrocytes from βA1 KD mice showed increased levels of NFκB-p65 phosphorylated at serine 536 (p-NFκB-p65^S536^), relative to WT and βA3 KO astrocytes (Fig. 6a, b). The serine 536 phosphorylation is known to be important for NFκB nuclear translocation^43^. Moreover, NFκB phosphorylation was increased in blank mCherry transfected βA1 KD astrocytes exposed to HG (30 mM for 6 h), compared to HG-exposed WT cells (Fig. 6c, d). However, βA1 KD astrocytes that were pre-transfected with the βA1-mCherry construct for 48 h or pre-treated with 10 μM of MSI-1436 for 1 h to inhibit PTP1B activity, and then exposed to HG, showed reduction in the levels of p- NFκB-p65^S536^, compared to blank mCherry transfected βA1 KD astrocytes (Fig. 6c, d). We also observed increased levels of IL-6 and IL-1α in the HG-exposed astrocytes from WT, βA3 KO and βA1 KD mice (Fig. 6e, f), which was also reduced by either βA1-crystallin overexpression or PTP1B activity inhibition (Fig. 6e, f). Interestingly, the increase in IL-6 and IL-1α levels in βA1 KD astrocytes treated with mannitol or HG (30 mM), for 6 h or left untreated, was noticeably higher relative to either WT or βA3 KO astrocytes (Fig. 6e, f). βA3 KO cells did not show any noticeable change in the levels of the two cytokines (IL-6 and IL-1α) when compared to WT astrocytes (Fig. 6e, f). These results indicate that a βA1-crystallin/PTP1B signaling axis regulates inflammatory signaling in astrocytes in hyperglycemic stress.

### βA1-crystallin protects against the onset of DR-like phenotype

**Fig. 7:**
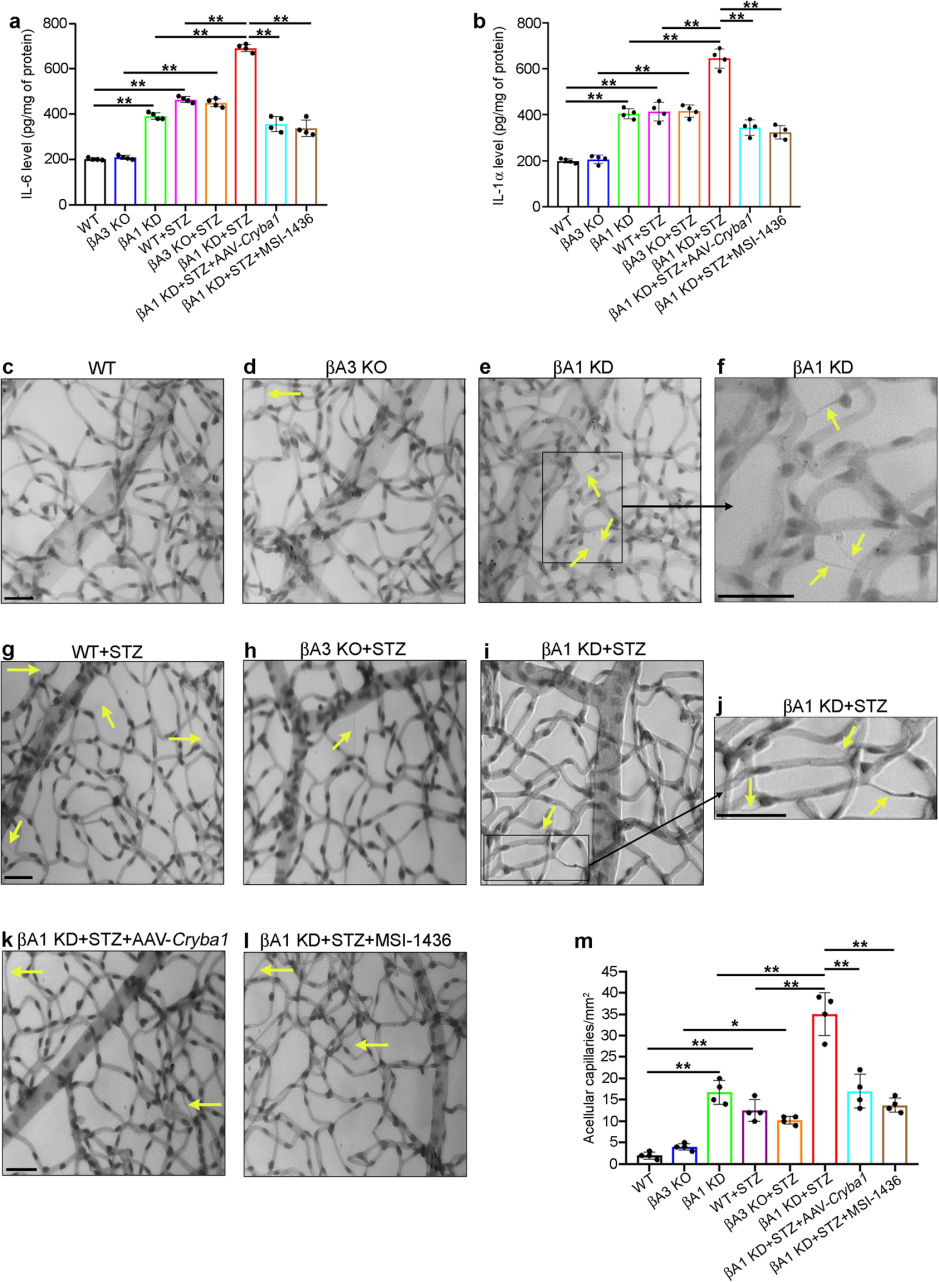
PTP1B and *Cryba1* are probable therapeutic targets for DR. ELISA analysis from total mouse retinal lysates showed increased **a** IL-6 and **b** IL-1α in diabetic (STZ-treated) WT, βA3 KO, and especially in βA1 KD mice relative to non-diabetic littermates. In diabetic βA1 KD mice, intraperitoneal treatment (thrice weekly) with PTP1B inhibitor (MSI-1436), at a dose of 0.125 mg/kg body weight started 3 weeks after diabetes onset and continued for 5 weeks or a single intravitreal injection of AAV2-*Cryba1* construct (1.64 × 10^12^ vg/ml), rescued the IL-6 and IL-1α levels. *n* = 4. **P < 0.01. **c**–**l** Representative images of the retinal capillary network and quantitative graph to show the number of acellular capillaries (**m**), in diabetic and non-diabetic WT, βA3 KO and βA1 KD mice, showing increase in acellular capillaries (arrows) in non-diabetic (**e**, inset zoomed in **f**) or diabetic βA1 KD mice (**i**, inset zoomed in **j**), compared to WT mice (**c**, **g**, and **m**). βA3 KO mice (**d**, **h**) did not show any noticeable change in retinal vasculature relative to WT mice. MSI-1436 or AAV2-*Cryba1* treatment reduced degenerative changes in the retinal vasculature (arrows in **k** and **l**, graph **m**) in βA1 KD animals. *n* = 4. *P < 0.05, **P < 0.01. Scale bar, 50 μm (**c**–**e**, **g**–**i**, **k**–**l**). Scale bar, 100 μm (insets, **f** and **j**).

Our results show that βA1 KD astrocytes are unable to metabolize glucose appropriately, thereby triggering ROS generation and inflammation. We postulate that this abnormality in βA1 KD astrocyte function during hyperglycemic stress could lead to alterations in the retinal vasculature and trigger pathologic remodeling of the retina similar to that seen in human DR patients^9^. To test this, we injected streptozotocin (STZ) intraperitoneally at a dose of 60 mg/kg body weight into 10-week-old WT, βA3 KO and βA1 KD mice to induce diabetes. Vehicle (citrate buffer) was administered to age-matched mice as a control. Induction of diabetes was confirmed by increased blood glucose and HbA_1c_ (hemoglobin A_1c_) levels and decreased body weight (Supplementary Fig. 2a–c) in the STZ injected animals, as previously explained^44^. Diabetic animals were maintained for another 2 months, and it was observed that there was increase in the retinal levels of IL-6 and IL-1α in the diabetic animals from all genotypes (Fig. 7a, b). While the levels of both cytokines in the diabetic βA3 KO retina were unchanged from those in diabetic WT retina, their levels in the retinas of both non-diabetic and diabetic βA1 KD mice were increased compared to WT and βA3 KO mice (Fig. 7a, b). Moreover, diabetic βA1 KD mice developed a DR-like phenotype (capillary degeneration) as evident from the increase in the number of acellular capillaries (Fig. 7m) in the retinas (arrows in Fig. 7i, j) and vascular leakage as shown by the prevalence of FITC-BSA fluorescence in the retinal tissue surrounding the blood vessels (arrows in Supplementary Fig. 3k, l), compared to diabetic WT and βA3 KO retina (Fig. 7g, h, m and Supplementary Fig. 3g–j). These are cardinal signals of DR-like phenotypes as seen in animal models of the disease^45,46^. Also, in comparison to non-diabetic WT and βA3 KO mice, we observed capillary degeneration (arrows in Fig. 7e, f) but no vascular leakage in non-diabetic βA1 KD mice (Supplementary Fig. 3e, f), indicating that this DR-like phenotype is further aggravated upon induction of diabetes. (Fig. 7i, j, m and Supplementary Fig. 3k, l). Only minimal indication of capillary degeneration or vascular leakage was observed in non-diabetic and diabetic WT or βA3 KO animals (Fig. 7c, d, g, h and Supplementary Fig. 3a–d, g–j), further demonstrating the importance of βA1-crystallin in maintaining retinal homeostasis in hyperglycemic stress.

Since these results suggest that βA1-crystallin regulates the PTP1B/STAT3 pathway during HG stress in retinal astrocytes, we postulated that inhibiting PTP1B systemically using the known PTP1B inhibitor, MSI-1436^47^, or overexpressing *Cryba1* in the retina by intravitreal injection of AAV2-*Cryba1* construct could alleviate the pathologic changes in the diabetic βA1 KD mice. We found that treatment with either MSI-1436 or AAV2-*Cryba1* construct for 2 months following diabetes onset did reduce IL-6 and IL-1α levels (Fig. 7a, b) as well as capillary degeneration (arrows in Fig. 7k, l).

### βA1-crystallin is decreased in the vitreous of human PDR patients

**Fig. 8:**
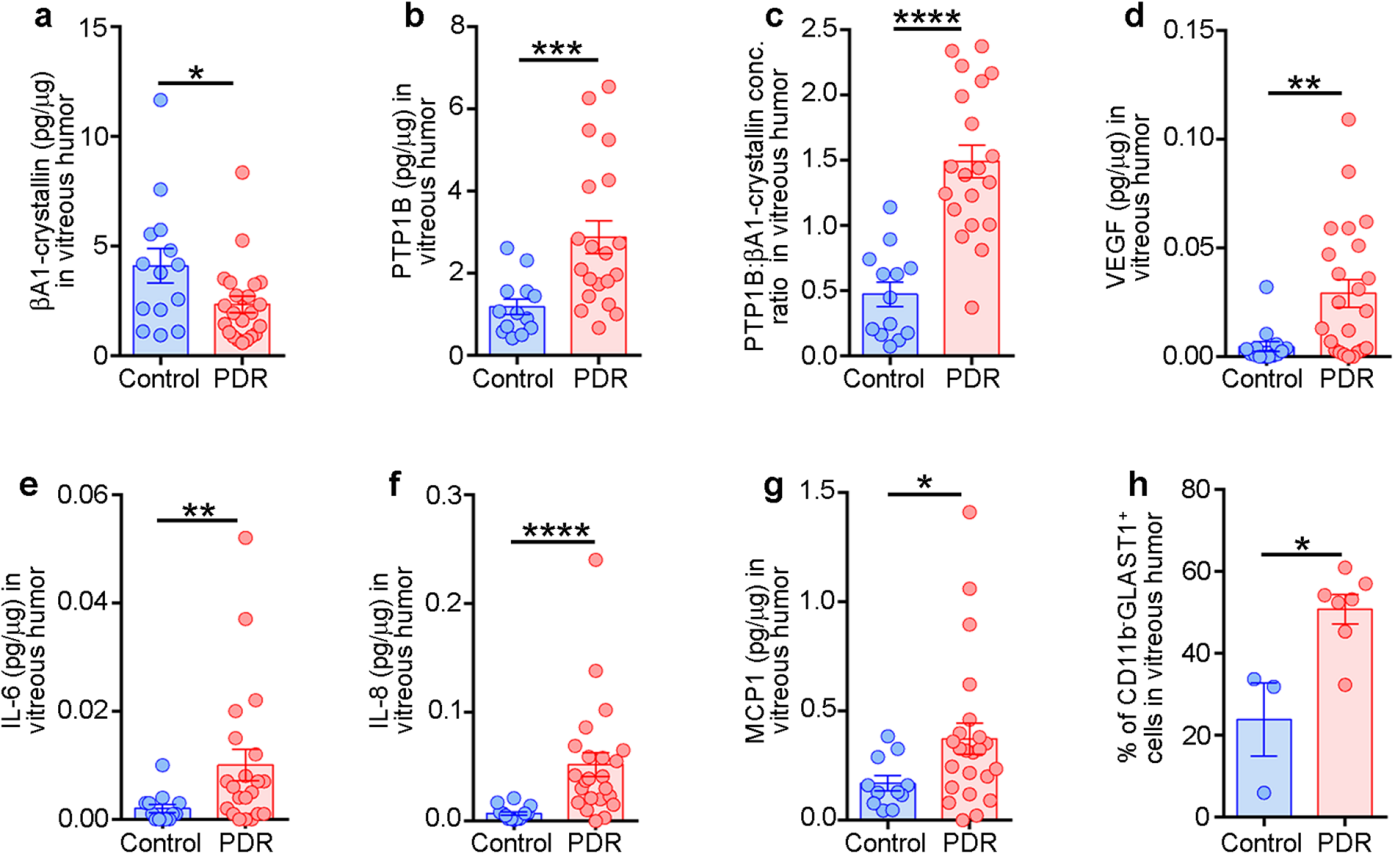
Altered levels of βA1-crystallin, PTP1B, VEGF, IL-6, IL-8, MCP1, and proportion of astrocytes in vitreous humor of patients with proliferative diabetic retinopathy. Graphs indicate the concentration of **a** βA1-crystallin, **b** PTP1B, **c** ratio of PTP1B and βA1-crystallin, **d** VEGF, **e** IL-6, **f** IL-8, **g** MCP1, and **h** proportion of astrocytes (CD11b^−^GLAST1^+^ cells) in the vitreous humor of patients with proliferative diabetic retinopathy (PDR) and control patients with macular hole (Control). The absolute concentration of each factor has been normalized to respective total protein concentration and represented as pg/μg. The sample size in panels **a**–**g** for control is *n* = 14 and for PDR is *n* = 23. The sample size in panel **h** for control is *n* = 3 and for PDR is *n* = 7. *P < 0.05, ***P < 0.001; ****P < 0.0001.

To provide clinical relevance, we obtained vitreous samples from human patients with PDR^48^, a pathology characterized by retinal vascular abnormalities culminating in pre-retinal neovascularization^48^. The levels of βA1-crystallin were reduced in these PDR patient samples (Supplementary Table 1), while PTP1B levels were notably higher in the same samples compared to control subjects without diabetes who had vitrectomy surgery for an unrelated macular hole (Supplementary Table 1 and Fig. 8a–c). Moreover, we also found that the expression of βA3/A1-crystallin in STZ-treated WT mice which were diabetic for 8 months, was reduced, compared to age-matched untreated WT mice (Supplementary Fig. 4a, b). Moreover, we found that the βA3 and βA1 isoforms showed 84%- and 96%-fold decrease in the diabetic retina, relative to the non-diabetic retina (Supplementary Fig. 4a, b). In addition, previous elegant studies have demonstrated that after 8 months of diabetes, WT mice exhibit DR-like pathology and increased inflammation in the retina^44,45^. We also found increased PTP1B activity in these diabetic retinas compared to the non-diabetic controls (Supplementary Fig. 4c). Therefore, these results further suggest that decreased expression of βA3/A1-crystallin is linked to an increase in PTP1B activity, which might be associated with DR pathogenesis. In addition to this, PDR patients who showed an altered ratio between βA1-crystallin and PTP1B also had ominously higher levels of soluble factors known to be critical for DR pathogenesis^49^ such as VEGF, IL-6, IL-8, and MCP1 in the vitreous humor compared to controls (Fig. 8d–g and Supplementary Table 2). It is important to note the positive association between the vitreous humor levels of VEGF, IL-8, and MCP1 with PTP1B, and the negative relationship between the vitreous humor levels of IL-6 and IL-8 and βA1-crystallin (Supplementary Table 2). This suggests the involvement of an interaction between βA1-crystallin and PTP1B in triggering retinal inflammation. Moreover, flow cytometry analysis of total cells in the vitreous from human samples (Supplementary Table 3) showed that there is an increased prevalence of CD11b^−^GLAST1^+^ astrocytes in human PDR patients compared to control subjects (Fig. 8h and Supplementary Fig. 5). GLAST1 is a known cell surface marker expressed on microglia and astrocytes^50^. To differentiate between the microglia and astrocytes in all of the samples, flow cytometry analyses were done, and microglia were identified as CD11b^+^GLAST1^−^ cells and astrocytes as CD11b^−^GLAST1^+^, as previously explained^51^.

**Fig. 9:**
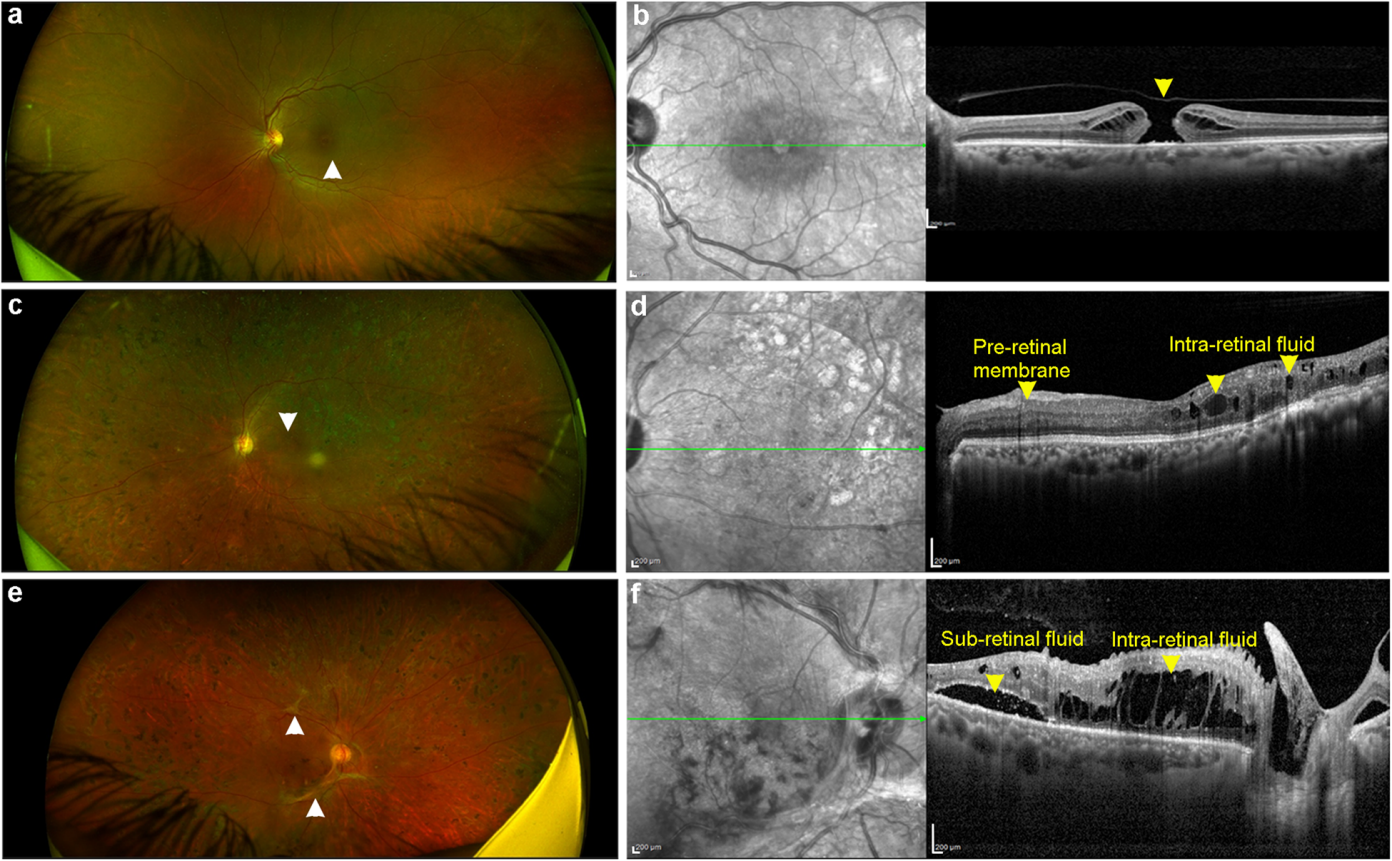
Representative fundus and spectral domain-optical coherence tomography images of subjects with macular hole or proliferative diabetic retinopathy. **a** Fundus image of full thickness macular hole (FTMH), indicated by a white arrow head. **b** Spectral domain-optical coherence tomography (SD-OCT) image of FTMH, indicated by a yellow arrow head. **c** Fundus image of a subject with post-lasered proliferative diabetic retinopathy (PDR) with macular edema, indicated by a white arrow head. **d** SD-OCT image of the eye indicated in panel **c** shows lasered PDR with intra-retinal fluid accumulation and pre-retinal membrane formation indicated by yellow arrow heads. **e** Fundus image of another subject with PDR with zones of fibrovascular proliferation indicated by white arrow heads. **f** SD-OCT image of the eye indicate in panel **e** shows presence of sub-retinal and intra-retinal fluid accumulation indicated by arrow heads. Scale bar, 200 μm (**b**, **d**, **f**).

In addition to this, clinical assessment of human PDR patients in the study cohort showed severe alterations in the retina. This was evident from fundus and optical coherence tomography (representative images; Fig. 9). OCT images clearly show sub and intra-retinal fluid accumulation (arrowheads in Fig. 9d, f) and pre-retinal membrane formation (arrowhead in Fig. 9d) compared to control subjects who had vitrectomy surgery for an unrelated macular hole (Fig. 9b). Fundus images of PDR patients also showed macular edema (arrowhead in Fig. 9c) along with zones of fibrovascular proliferation (arrowhead in Fig. 9e), which are cardinal features of PDR^52,53^.

## Discussion

We have previously shown that βA3/A1-crystallin has regulatory function in retinal astrocytes and that loss of functional βA3/A1-crystallin leads to alterations in retinal vasculature^10,16,54^. Our studies^10,19,54^ have also suggested that βA3/A1-crystallin may be considered as a “moonlighting protein,” a class of proteins where a single protein performs multiple physiologically relevant biochemical or biophysical functions^55^. It has been predicted that when translation is initiated using alternative start sites (βA3-crystallin has 17 additional N-terminal amino acid residues)^1^, the isoforms may be delivered to different subcellular compartments and have distinct functions. In the case of βA3/A1-crystallin, the possibility of different roles for the individual isoforms (βA3 and βA1) in regulating distinct cellular functions has received little attention.

Here we show the functional distinction between two crystallin isoforms in regulating PTP1B-dependent glucose metabolism in retinal astrocytes. PTP1B is a phosphatase that is ubiquitously expressed and is a known negative regulator of the leptin and insulin signaling pathways^6^. Increased PTP1B phosphatase activity has been implicated in several disease processes, including DR^7,8^. βA1-crystallin inhibits PTP1B activity and regulates STAT3 signaling in astrocytes, while the βA3 isoform has little or no such effect. PTP1B is known to negatively regulate the Jak2/STAT3 signaling pathway^29^, which is critical in regulating glucose metabolism^30,31^ and has been linked to metabolic diseases^56,57^. We have previously shown that βA3/A1-crystallin is necessary for the phosphorylation of STAT3 and its translocation into the nucleus, where it activates transcription of various genes including *Cryba1* as part of a positive feed-forward loop^32^. We now find that the loss of βA1-crystallin resulted in elevated PTP1B activity, thereby causing a decrease in STAT3 phosphorylation and its nuclear localization. These alterations trigger abnormalities in oxidative phosphorylation and ATP generation thereby causing oxidative stress. Mitochondria are central to cellular metabolism, are major regulators of redox balance, and play a crucial role in the pathogenesis of many diseases^58,59^. It is known that a decrease in mitochondrial respiration and glucose utilization increases mitochondrial ROS production, raising cellular ROS levels^25^. We find definitive metabolic changes in astrocytes when βA1-crystallin is knocked down, but complete loss of βA3-crystallin does not cause such changes, perhaps due at least in part to compensation by increased synthesis of βA1-crystallin. This demonstrates that βA1-crystallin is the major isoform involved in glucose metabolism in astrocytes. We show that βA1-crystallin is essential in astrocytes for mitochondrial respiration and maintenance of cellular energy homeostasis.

The role of astrocytes in DR pathogenesis is still uncertain. Previous reports have suggested that hyperglycemic stress triggers alterations in astrocyte function in DR^42,60^ and other diabetes-associated disorders such as diabetic neuropathy^13^, but the underlying signaling associated with astrocyte dysfunction in hyperglycemic stress remains largely unknown. We show here that in the diabetic βA1 KD mouse there is activation of inflammatory signaling pathways, leading to the release of a plethora of free radicals and pro-inflammatory molecules into the surrounding tissue, probably potentiating a DR-like phenotype (capillary degeneration and vascular leakage). The metabolic changes observed with the loss of βA1-crystallin appear to be exacerbated under hyperglycemia, which could suggest an involvement in the regulation of DR pathology and requires further investigation. Also, we found increased phosphorylation of NFκB at serine 536, which is known to be important for NFκB nuclear translocation^43^ and elevated levels of IL-6 and IL-1α in βA1 KD astrocytes, probably owing to the intrinsic abnormality in mitochondrial function along with the elevated mROS in these astrocytes^40^. However, to pinpoint the effect of hyperglycemia on the involvement of either NFκB-dependent or other intracellular signaling cascades (if any) in potentiating the activation of inflammation in these retinal astrocytes, needs further experimental validation. In addition, our results also link metabolic changes in the astrocytes to immune dysregulation in the retina. Previous studies showing that increased expression of IL-6 and IL-1α is associated with vascular abnormalities in animal models of DR^61^ and in macular edema in human DR patients^62^ are similar to our findings. Our studies suggest that astrocytes are probably a major contributor to pathological remodeling of the retina. βA3/A1-crystallin is also expressed in the retinal pigmented epithelial (RPE) cells^63^, but we have not observed nuclear localization of either isoform in RPE cells and furthermore, in mice where βA3/A1-crystallin is conditionally knocked out in the RPE, no vascular abnormalities are observed. Thus, we have not considered the possible influence of RPE cells in remodeling of the retina.

Interestingly, DR is a disease that is now more accurately defined as a neurodegenerative disease that precedes and coexists with vascular changes^64^. The developmental function of astrocytes in organizing the retinal vasculature expands into their postnatal role in maintaining retinal homeostasis, where they are known to provide trophic support to retinal ganglion cells as well as helping to maintain vascular integrity^65,66^. Taken together, it is highly likely that loss of βA1-crystallin function in astrocytes triggers pathological changes similar to DR. Fully defining such a possibility will require further in-depth studies.

One of the notable observations in our study was that vitreous samples obtained from human PDR patients showed increased numbers of astrocytes (CD11b^−^Glast1^+^). Presence of GFAP, a marker for glial cells including astrocytes in the vitreous humor homogenates of human cadaveric donor controls and subjects with macular hole or epiretinal membrane suggests the presence of astrocytes in normal vitreous humor^67,68^. Interestingly, we have shown previously that astrocytes lacking functional βA3/A1-crystallin proliferate and migrate abnormally into the vitreous and ensheath the hyaloid artery, thereby contributing to persistent fetal vasculature (PFV) disease^69^. However, it has been previously shown that the GFAP levels were higher in subjects with retinal detachment, proliferative vitreoretinopathy or epiretinal gliosis^67,68^. Studies in rabbits have demonstrated that the astrocytes are separated from the vitreous by a thin basement membrane of the inner limiting membrane and any disruption or gaps in the latter would allow the contact between astrocytes and vitreous body^70^, thereby suggesting that astrocyte numbers would increase in the vitreous humor of an eye with retinal disease such as DR. Further, we observed that DR patients with the highest PTP1B/βA1 ratios also had higher levels of pro-inflammatory factors in their vitreous. We therefore propose that βA1-crystallin regulates glucose metabolism in retinal astrocytes by modulating PTP1B activity and that the loss of this regulation could induce retinal inflammation and might exacerbate DR-like pathology. Moreover, previous published studies have also showed a decrease in the levels of βA3/A1-crystallin in vitreous of DR patients^71,72^, while glial fibrillary acidic protein (GFAP; a known astrocyte marker) is upregulated in the vitreous of patients suffering from various retinal diseases^67^.

**Fig. 10:**
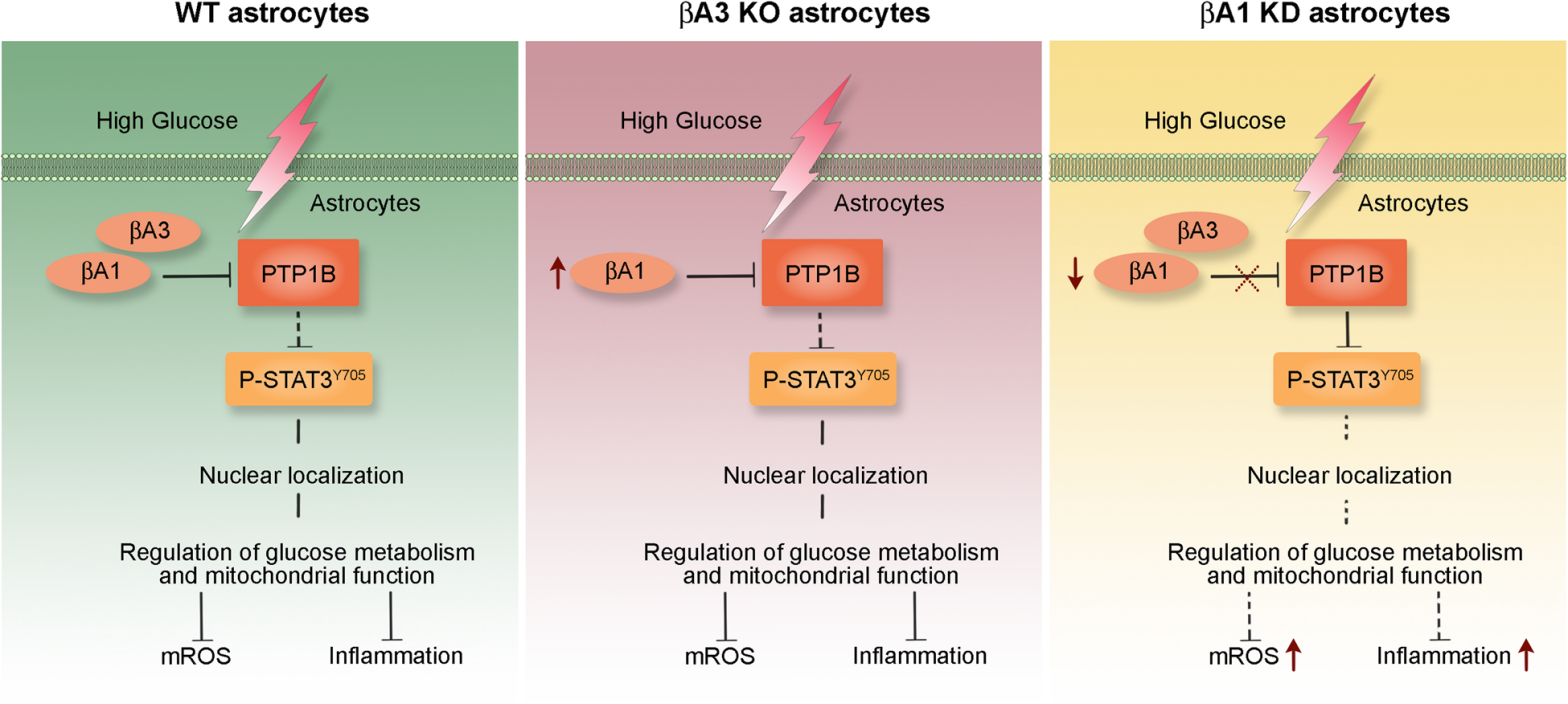
βA1-crystallin regulates PTP1B/STAT3 signaling axis in retinal astrocytes. High glucose exposure to WT and βA3 KO astrocytes produce no deleterious effect due to the appropriate regulation of PTP1B-dependent STAT3 signaling and glucose metabolism by the presence of βA1-crystallin in these cells. However, knockdown of βA1-crystallin, triggers abnormally high PTP1B activity, thereby inhibiting the downstream STAT3 signaling and glucose metabolism, causing oxidative stress and inflammation in βA1 KD cells. These changes in astrocytes might trigger DR-like pathology in the βA1 KD mice in diabetic conditions. Taken together, and to the best of our knowledge, this study provides evidence that supports the role of βA1-crystallin in maintaining retinal homeostasis in hyperglycemic stress and offers a possible target for the treatment of DR.

Here, we show that βA1-crystallin regulates PTP1B activity in retinal astrocytes, owing to its ability to allosterically inhibit PTP1B phosphatase activity (Fig. 10). PTP1B links metabolism and inflammation in diabetes^5^. Our findings suggest that in βA1-crystallin knockdown mice injected with STZ exacerbates a DR-like phenotype. However, to determine whether βA1-crystallin is a cause or effect of DR, further investigations are needed. We speculate that targeting the βA1-crystallin/PTP1B axis could be a probable therapeutic approach for the treatment of DR.

## Methods

### Reagents

Primary antibodies: Phospho-Stat3 (Tyr705) (Thermo Fisher, USA; Cat# 44380G), p-NFκB p65 (S536) (Thermo Fisher, USA; Cat# MA515160), STAT3 (Thermo Fisher, USA; Cat# 10253-2-AP), beta Crystallin A3 (Abcam, USA; Cat# ab151722), IL-6 (Biorbyt, USA; Cat# orb6210), IL-1α (Biorbyt, USA; Cat# orb184287) and mNeonGreen (Chromotek, USA; Cat# 3216-100), Secondary antibodies: HRP anti-Rabbit IgG (KPL, USA; Cat# 074-1506), HRP anti-tagged anti-Mouse IgG (KPL, USA; Cat# 5220-0341), HRP anti-tagged Goat IgG (KPL, USA; Cat# 14-13-06). GIPZ *Cryba1* shRNA Viral Particle Starter Kit (Dharmacon, USA; Cat# VGH5526-EG12957), AAV2-GFP-U6-shRNA (Vector Biolabs, USA; Cat# 7041). PTP1B (Ad-CMV-mNeonGreen-m*Ptpn1*; Cat# AAV-269791) and *Cryba1* (Ad-CMV-RFP-m*Cryba1*; Cat# 2001) overexpression vectors were purchased from Vector Biolabs, USA. ELISA kits: Human crystallin, beta A1 (CRYBA1) (Cat# MBS7252187) and protein tyrosine phosphatase 1B (PTP1B) (Cat# MBS761801) ELISA kits were purchased from Mybiosource, USA. Proteins: CRYBA1 (NM_005208) purified human protein (OriGene Technologies, Cat# TP321965), Recombinant human PTP1B protein (Abcam, Cat# ab51277).

### Animals

All animal studies were conducted in accordance with the Guide for the Care and Use of Animals (National Academy Press) and were approved by the University of Pittsburgh Animal Care and Use Committee. Sprague Dawley (WT) and Nuc1 rats were maintained as explained earlier^19,32^. βA3 KO and βA1 KD mice were generated in C57BL6J background by using CRISPR/Cas9 genome editing through modifying the sequence of *Cryba1* to affect relative translation of the βA3- and βA1-crystallin proteins. To eliminate the expression of the longer βA3-crystallin protein, the first start codon was abolished by a single nucleotide replacement (m*Cryba1* [A > G] mutation knockin). A silent mutation (ACC to ACG) was also introduced to prevent the binding and re-cutting of the sequence by gRNA after homology-directed repair. To reduce the expression of βA1-crystallin without affecting the protein sequence, 5 base pairs were knocked-in before the first start codon to strengthen the Kozak consensus sequence (CCACCATGG). The mutations were confirmed by Sanger sequencing using DNA sequencing primer (forward sequencing) 5′-CCCCAATAGGCTGAGCCACTAAAG-3′ for A to G mutation and 5′-CCTGCACTTCTGGAACCCTAAACA-3′ for the CCACC mutation. The gRNA/Cas9 mRNA and donor DNA with the mutations were injected into the fertilized eggs from C57BL6N mice to generate correct targeted F0 founders. Mouse lines were generated at Cyagen, Inc. carrying these modified genes, and individuals homozygous for the modified genes were produced by selective mating. PCR genotyping was performed using the following primers; mCryba1-F: 5′CCTGCACTTCTGGAACCCTAAACA-3′ and mCryba1-R: 5′TAGCAAATGAAGCTGTCCCCCAC-3′. The mice were originally bred into the C57BL/6N strain, which carries the rd8 mutation, but this retinal degeneration mutation was bred out of the colony before this study was conducted.

### Astrocyte culture

Two-day-old pups from WT and Nuc1 rats along with WT, βA1 KD, and βA3 KO mice were cultured as previously described^19,32^. High-glucose treatment was provided after the cells reached a confluency of about 80% by adding 25^42,60^ or 30 mM^73,74^ d-Glucose (Sigma Aldrich, USA; Cat# G8644-100ML) for 6 h depending on the experimental requirement. For osmolarity control, cells received mannitol (Sigma Aldrich, USA; Cat# M4125-100G) at a dose of 25 or 30 mM for 6 h. Untreated cells (controls) were grown in media containing 5 mM d-Glucose.

### Generation of βA1 and βA3 constructs

RNA was extracted with TRIzol (Invitrogen, Carlsbad, CA) from a mouse lens, and cDNA was made using SuperScript™ VILO™ cDNA Synthesis Kit (Invitrogen, USA; Cat# 11754-050). *Cryba1* was amplified by PCR using High-Fidelity Phusion polymerase (Thermo Scientific, USA; Cat# F530L). The primers contained EcoRI and XhoI restriction enzyme sites. mCherry sequence (without the start codon) and the upstream flexible linker were amplified by PCR from LIC-mCherry vector (Addgene, USA; plasmid #30125) using High-Fidelity Phusion polymerase. The primers contained XhoI and XbaI restriction enzyme sites. EcoRI-Cryba1-XhoI and XhoI-linker-mCherry-XbaI PCR products were gel purified using Wizard® SV Gel and PCR Clean-Up System (Promega, USA; Cat# A9282) and ligated into the pCR-Blunt vectors (Invitrogen, USA). The linker-mCherry was then excised from the Zeroblunt vector using XhoI and XbaI double digest (FastDigest Restriction Enzymes, Thermo Scientific, USA) and ligated into pcDNA3.1(+) vector (Invitrogen, USA). This resulting mCherry/pcDNA3.1(+) vector was linearized with EcoRI and XhoI and ligated with *Cryba1* that had been excised from the Zeroblunt vector using EcoRI and XhoI double digest. All the digested fragments were gel purified prior to ligation. Finally, QuikChange Lightning Site-Directed Mutagenesis kit (Agilent Technologies, USA; Cat# 210515) was used to mutate the first codon of *Cryba1*, to introduce a silent mutation in the third codon of *Cryba1*, and to mutate the stop codon of *Cryba1* to ensure continuous translation of the Cryba1-linker-mCherry sequence. The presence of the mutations was confirmed by sequencing performed by Genewiz (South Plainfield, USA).

### Cell transfection and PTP1B inhibition in vitro

Transfection of astrocytes with βA1- and βA3-crystallin vectors was performed using Neon^TM^ Transfection system according to manufacturer’s protocol (Thermo Fisher, USA; Cat# MPK10096). For appropriate transfection, cells were cultured for 48 h in medium containing 5 mM d-Glucose and then the medium was replaced with high glucose (30 mM) containing medium for another 6 h. WT astrocytes were infected with either GIPZ *Cryba1* shRNA or PTPN1 (Ad-CMV-mNeonGreen-m*Ptpn1*) overexpression construct for 48 h, and then high glucose medium was added to the cells for the next 6 h. For PTP1B inhibition, MSI-1436 (MedChemExpress, USA; Cat# HY-12219A) was added to βA1 KD astrocytes in culture at a dose of 10 μM^24^, 1 h prior to addition of high glucose.

### Live cell imaging

Human iPSC-derived astrocytes (Tempo Bioscience, USA; Cat# SKU101) were transfected with βA1-mCherry or βA3-mCherry constructs. The cells were mounted in 35 mm glass bottomed dishes (Mattek Corporation) in a Tokai Hit environmental chamber (Tokai Hit, Tokyo, Japan) at 37 °C and imaged using a Nikon Ti microscope and Sweptfield confocal scan head (Nikon, Tokyo, Japan). In total, 30 mM glucose was added immediately prior to imaging. Three-dimensional image stacks were collected every 10 min from 12 to 14 fields in each dish for up to 17 h. The 3D stacks were deconvolved using a Richardson Lucy algorithm (NIS Elements) and the midplane image from each stack selected as a time series.

Image quantification was performed on deconvoluted images using ImageJ software. Mean intensities were measured by setting up region of interests for total cell, nuclear, and background signals. The background was subtracted from each value (total and nuclear), whereas the cytosolic signal was obtained by subtracting nuclear mean intensities from total cell intensities. The cytosolic and nuclear intensity values were normalized with respect to the area. The nuclear and cytosolic ratio was obtained for each experimental condition to deduce the nuclear localization of either crystallin isoforms.

### PTP1B activity

PTP1B activity was measured in astrocytes using p-nitrophenyl phosphate (pNPP) as substrate according to a previously published protocol^75^. Cells were lysed, and retinal tissue was homogenized in lysis buffer (50 mM Bis-Tris, 2 mM EDTA, 0.1 mM PMSF, 5 mM DTT, 0.5% Triton X-100, and 20% glycerol) on ice, and total protein was determined using BCA Assay Kit (Thermo Fisher, USA). Samples were diluted to 1 μg/μL using dilution buffer (50 mM Bis-Tris, 2 mM EDTA, 5 mM DTT, and 20% glycerol). In all, 175 μL of assay buffer (50 mM Bis-Tris, 2 mM EDTA, 5 mM DTT, 0.01% Triton X-100, and 15 mM p-nitrophenyl phosphate) was added to each sample and incubated for 30 min at 37 °C. Reaction was stopped by addition of 20 μL of 10 M NaOH, and the absorption was measured at 405 nm after 5 min.

### PTP1B inhibition

The kinetic analysis to evaluate the potency of βA3/A1-crystallin as an inhibitor of PTP1B was performed by making reaction mixtures consisted of three different concentrations of pNPP (1.0, 2.0, and 8.0 mM) with 0.1 μg of recombinant human PTP1B protein (Abcam, USA; Cat# ab51277) in the presence of different amounts (0, 0.5, 0.75, 1, and 2 nM) of βA3/A1-crystallin (NM_005208) purified human protein (OriGene, USA; Cat# TP321965). The Michaelis−Menten constant (*K*_m_) and maximum velocity (*V*_max_) of PTP1B were plotted as Lineweaver−Burk plots using MS Excel.

### Human study cohort

The cross-sectional study following approval by the Narayana Nethralaya Institutional Review Board was performed as per guidelines stipulated by the Indian Council for Medical Research (ICMR). Subjects were recruited for the study post informed written consent as per institutional and ethics board guidelines and as referred to Narayana Nethralaya Eye Hospital, Bangalore, India. The inclusion and exclusion criteria for the study are as follows: inclusion criteria: (i) patients with proliferative diabetic retinopathy (PDR) confirmed with vascular proliferation at macula as diagnosed based on fundus imaging, OCT, and FFA. (ii) Subjects undergoing surgical intervention (as part of standard of care) that would require access into vitreous humor. Exclusion Criteria: (i) PDR patients with additional complications such as tractional retinal detachment and vitreous hemorrhage. Patients without any sign of vascular proliferation/abnormality in the retina but having other retinal conditions such as macular hole and floaters were scheduled for vitrectomy as a part of standard care and were considered as control. Vitreous humor (VH) samples were collected from age-matched 14 controls (macular hole, *n* = 11; floaters, *n* = 3; age; M/F – 7/7) and 23 PDR subjects (M/F – 17/6) for the measurement of βA1-crystallin, PTP1B, VEGF, IL-6, IL-8, and MCP1 (Supplementary Table 1). Vitreous humor (VH) samples were also collected from age-matched 3 controls (macular hole, *n* = 3; M/F – 1/2) and 7 PDR subjects (M/F – 5/2) for determining the proportion of astrocytes measurement (Supplementary Table 3). Collection of VH was performed by the vitreo-retinal surgeon at the beginning of three-port pars plana vitrectomy as per standard of care procedure. All the samples were stored at −80 °C until further processing.

### Measurement of βA1-crystallin, PTP1B, pro-angiogenic, and pro-inflammatory factors in vitreous humor

Levels of βA1-crystallin and PTP1B in VH were measured by ELISA (βA1-crystallin: MyBiosource, USA; Cat# MBS7252187 and PTP1B: MyBiosource, USA; Cat# MBS761801) as per manufacturer’s instruction. Briefly, VH samples were centrifuged at 400 g for 15 min. The supernatant was homogenized by repeat pipetting and pulse vortexing (2 s X 5 times). Required volume of VH was diluted with 1X phosphate buffered saline (pH 7.4) or sample diluent, and ELISA was performed as per the manufacturer’s instructions. Absolute concentrations were derived based on standard curve. The levels of VEGF, IL-6, IL-8, and MCP1 in VH were measured by bead-based ELISA (Cytometric Bead Array, BDTM CBA Human Soluble Protein Flex Set System, BD Biosciences, USA) using a flow cytometer (BD FACS Canto II, BD Biosciences, USA). The beads and fluorescent signal intensities were acquired and recorded using BD FACSDiva software (BD Biosciences, USA). Standards were used to determine the absolute concentration of the analytes, and the calculations were performed using FCAP array Version 3.0 (BD Biosciences, USA). Total protein concentration in the VH was estimated by BCA assay (G-Biosciences) as per manufacturer’s protocol. The absolute concentrations (pg/ml) of the measured factors were normalized to the total protein concentrations (μg/ml) of the respective samples.

### Quantification of astrocyte proportions in the vitreous humor

The proportions of astrocytes in vitreous humor (VH) of subjects were determined by flow cytometry-based phenotyping using fluorescence-conjugated antibody specific to astrocytes. Extracellular epitope of the astrocyte-specific l-glutamate/l-aspartate transporter 1 (GLAST1) has been used to phenotype and isolate astrocytes^76^. Further, cells positive for GLAST1 and negative for CD11b (phenotypic marker for microglia) were considered to be specific astrocyte population. Briefly, VH samples were centrifuged at 400 g for 15 min, and the cell pellet was stained with antibody cocktails diluted in staining buffer (5% Fetal Bovine Serum in 1X Phosphate Buffer Saline, pH 7.4) by agitation (500 rpm) for 45 min at room temperature. Antibody cocktails include fluorochrome conjugated CD11b (BV510, *clone ICRF44*, BD Biosciences, USA; Cat# 563088) and GLAST1 (PE, *clone ASCA-1*, Miltenyi Biotec, USA; Cat# 130-118-344). Data acquisition was done on a flow cytometer (BD FACS Lyric, BD Biosciences, USA) and analysed using FCS Express 6 (De Novo Software, USA). Post-acquisition compensation for the flowcytometry data was done using single stained controls. Manual gating strategy was applied to identify astrocyte population and is shown in Supplementary Fig. 4.

### ELISA

Retinal tissue from diabetic and non-diabetic WT, βA3 KO, and βA1 KD mice was homogenized in 200 μL of complete extraction buffer (Abcam, USA; Cat# ab193970), kept on ice for 20 min, and centrifuged at 10,000 rpm for 20 min at 4 °C. WT, βA3 KO, and βA1 KD astrocytes harvested from culture were suspended in 100 μL of complete extraction buffer, kept on ice, and then sonicated. The lysates were used to perform Enzyme-linked immunosorbent assay (ELISA) in 96-well microtiter plates as previously described^77^.

### Seahorse experiment

Metabolic flux in astrocytes from different genotypes was evaluated by using the glycolytic stress (Cat# 103020-100) and the Mitostress assay (Cat# 103015-100) kits from Agilent, USA. Cells were plated on a Seahorse XF platform compatible 96-well plate pre-coated with poly-D-lysine (Sigma Aldrich, USA) at 40,000 cells per well and allowed to adhere and grow for 24 h. Transfection (see above) with mCherry-βA1 construct or treatment with MSI-1436 (10 μM) in βA1 KD astrocytes were performed for 48 h and 1 h respectively, prior to HG (30 mM) treatment for 6 h. The assays were then performed according to the manufacturer’s protocol.

### Mitochondrial ROS

WT, βA3 KO, and βA1 astrocytes either untreated or cultured in presence of 30 mM HG or mannitol respectively, for 6 h. Transfection (see above) with mCherry-βA1 construct or treatment with MSI-1436 (10 μM)^24^ in βA1 KD astrocytes was performed for 48 h and 1 h, respectively, prior to HG (30 mM) treatment for 6 h. Cells from all experimental conditions were washed with PBS and incubated with 5 μM MitoSOX (Thermo Fisher, USA; Cat# M36008) for 15 min at 37 °C, protected from light. The cells were washed with PBS thrice, scraped from the plate, and analysed using a flow cytometer (BD FACS Aria III). The data was analyzed using FlowJo software (v10.6.1)^78^.

### Superoxide release

To freshly isolated astrocyte-spent media from different experimental groups, lucigenin was added at a final concentration of 0.5 mM. After 5 min, luminescence was measured using Glomax bioluminescence apparatus (Promega, USA; Cat# E5311) as previously described^44^.

### Diabetes induction and analysis

To induce diabetes in 10-week-old WT, βA3 KO, and βA1 KD mice, the animals were fasted for 6 h, and STZ was given intraperitoneally at a dose of 60 mg/kg body weight as previously described^79^. Diabetes was defined by non-fasted blood glucose concentrations greater than 250 mg/dl, which was verified using glucose-dehydrogenase-based strips (Harmony blood glucose strips, Medline, USA; Cat# MPH6550Z) on three consecutive occasions beginning 1 week after the STZ injection; hyperglycemia was also quantified by hemoglobin A1c (HbA1c) levels using the mouse HbA1c kit (Crystal Chemical, USA; Cat# 80310) and body weight^79^. Animals were euthanized after two months in diabetic condition.

### Vascular permeability

WT, βA3 KO, and βA1 KD mice were injected FITC-BSA (50 μg/μl; Sigma-Aldrich; St Louis, MO, USA) in PBS (NaCl, 0.138 M; KCl, 0.0027 M; pH 7.4) was injected into the tail vein of mice at 100 μg/g body weight^80^. After 30 min, animals were euthanized and eyes were enucleated immediately and fixed in 2% paraformaldehyde (PFA) for 10 min. The anterior parts, including cornea, lens, and attached iris pigmented epithelium, were removed. The resulting posterior eyecups were fixed in 2% PFA for 1 h at room temperature for neural retina flat mounts prepared as described previously^75^. Neural retina flat mounts were mounted on microscope slides. Images were acquired by Zeiss LSM 710 confocal microscope (Zeiss, Switzerland).

### MSI-1436 and AAV2-*Cryba1* vector

βA1 KD mice were made diabetic with STZ and were given either intraperitoneal (thrice weekly)^47^ or intravitreal^77^ (one time) injections of 0.125 mg/kg body weight of MSI-1436^47^ or 1.64 × 10^12^ vg/μl of AAV2-*Cryba1* (Lot# 170123#05, Vector Biolabs, USA) vector, respectively, three weeks after onset of diabetes. Experiments were performed on these animals 5 weeks after the first injection. All instruments were autoclave-sterilized. Bacitracin ophthalmic ointment was applied after intravitreal injection.

### Western blotting

Western blotting was performed as previously described^4,77^. Quantification was performed after normalization with loading control and evaluating the signal intensity of protein bands using ImageJ^4,77^. The values were normalized to a housekeeping protein. All targeted proteins and loading controls were verified to be in a linear range.

### Quantitative PCR

qRT-PCR was performed as previously described^4^ using Taqman probes (Life Technologies, USA) for mouse *Nox1* (Cat# Mm00549170_m1), Nox2 (Cat# Mm00516005_m1), *Lepr* (Cat# Mm00440181_m1), and *Jak2* (Cat# Mm01208489_m1).

### Single-cell RNA-sequencing and analysis

Mixed primary cultures of glia cells from the optic nerve of WT and Nuc1 rats were prepared as described previously^81^. Single-cell RNA sequencing (scRNA-seq) was performed as a paid service from Genomics Research Core of University of Pittsburgh to identify the RNA expression profile of astrocytes. R package Seurat 3.1.0^82^ was used to do an integrated analysis for performing clustering analysis after merging WT cells and Nuc1 cells together. Data was demultiplexed using CellRanger Software (version 3.1.0) from 10x Genomics, and paired end FASTQ files were generated. The reads were aligned to the reference genome using STAR aligner (version 2.7)^83^, and the alignment results were used to quantify the expression level of genes. Downstream analysis was done using Seurat (version 3.1.1)^84^. Data were pre-filtered to remove low quality cells, doublets, and potentially dead cells based on the percentage of mitochondrial genes, number of genes, and Unique Molecular Identifiers (UMI’s) expressed in each cell. The standard pipeline of normalization such as finding variable features, scaling, and dimensionality reduction by principal component analysis was followed. The integrated dataset of the WT and Nuc1 samples was then used for the UMAP clustering and differential gene expression analysis.

### Co-immunoprecipitation

WT and *Cryba1* KO mouse astrocytes were cultured up to 80% confluency and were transfected with pcDNA-βA3-mCherry, pcDNA-βA1-mCherry, pcDNA-βA3/A1-mCherry, and pcDNA-mCherry (as explained above) for 48 h and lysed in RIPA buffer supplemented with protease inhibitors cocktail (Sigma Aldrich, USA; Cat# I3786-1ML). Cell extracts were incubated with RFP-magnetic beads (Chromotek, USA; Cat# rtmak-20) for 2 h at 4 °C. Collected beads were washed with washing buffer (10 mM Tris/Cl pH 7.5, 150 mM NaCl, 0.5 mM EDTA, and 0.018 % sodium azide) and eluted with 2x SDS-sample buffer. The eluted samples were analyzed in a SDS-PAGE gel followed by western blot to assess the binding of PTP1B. WT astrocytes were co-infected with Ad-CMV-mNeonGreen-m*Ptpn1* and Ad-CMV-RFP-m*Cryba1* constructs to overexpress PTP1B and βA3/A1-crystallin. The RFP in the *Cryba1* construct is expressed under a separate promoter. The mNeonGreen-m*Ptpn1* (PTP1B) pull down assay from astrocyte lysates was performed by preparing anti-mNeonGreen and mouse IgG magnetic beads using the manufacturer’s protocol (Dynabeads Co-Immunoprecipitation Kit, Thermo Fisher, USA; Cat# 14321D). The pull down was followed by western blotting for βA3/A1-crystallin in the Co-IP eluent.

### Molecular modeling

The possible interaction between βA3-crystallin or βA1-crystallin and PTP1B was tested using an interactive protein docking and molecular superposition program HEX PROTEIN DOCKING, version 6.3 as explained previously^20^.

### Retinal vasculature

Acellular capillaries were quantified in the retinal vasculature as previously described^80^. Freshly enucleated eyes from WT, βA3 KO, and βA1 KD mice from different treatment groups were fixed with 10% formalin. Retinas were incubated in elastase for 2 h followed by incubation in Tris buffer (pH 8.5) overnight. Retinal vasculature was stained with hematoxylin and periodic acid-Schiff. Acellular capillaries were quantified in 7 field areas between the optic nerve and the periphery and then quantified as previously described^80^.

### Statistics and reproducibility

For experiments involving human samples, Shapiro–Wilk normality test was performed to determine the distribution of the data. Mann-Whitney test and Spearman Rank Correlation analysis were done to determine the difference in parameters between the test groups and the association between the factors. Statistical analyses were performed with either GraphPad Prism 6.0 (GraphPad Software, Inc., La Jolla, CA, USA) or MedCalc® Version 12.5 (MedCalc Software bvba, Ostend, Belgium). *P* < 0.05 was considered statistically significant^77^. For animal and cell culture experiments, the analyses were performed on triplicate technical replicates. The number of biological replicates (*n*) for each experiment is mentioned in the respective figure legend. The statistical analysis was performed with Microsoft Excel and GraphPad Prism 8 software for Mac using one-way ANOVA. Group means were compared using Tukey’s post hoc test with significance being set at *P* < 0.05^77^. All results are presented as mean ± standard deviation (SD) or standard error of mean (SEM) as indicated in the figure legends.

### Reporting summary

Further information on research design is available in the Nature Research Reporting Summary linked to this article.

## Supplementary information

Supplementary Information

Description of Supplementary Files

Supplementary Movie 1

Supplementary Data 1

Reporting Summary

## Data Availability

All data generated or analyzed during this study are included in this published article (and its Supplementary files). The raw and analyzed data files for the single-cell RNAseq (scRNAseq) experiment can be accessed in Geo NCBI database (Accession No: GSE159452). Source data for figures can be found in Supplementary Data 1. Uncropped blot/gel images are shown in Supplementary Fig. 6. All relevant data relating to this manuscript are available upon request.
