# Supplementary material for: βA1-crystallin regulates glucose metabolism and mitochondrial function in mouse retinal astrocytes by modulating PTP1B activity
Source: Commun Biol. 2021 Feb 24;4:248. doi: 10.1038/s42003-021-01763-5 (PMC7904954; doi:10.1038/s42003-021-01763-5)
Supplement: Supplementary file 1 — Supplementary Information [file 42003_2021_1763_MOESM1_ESM.pdf]

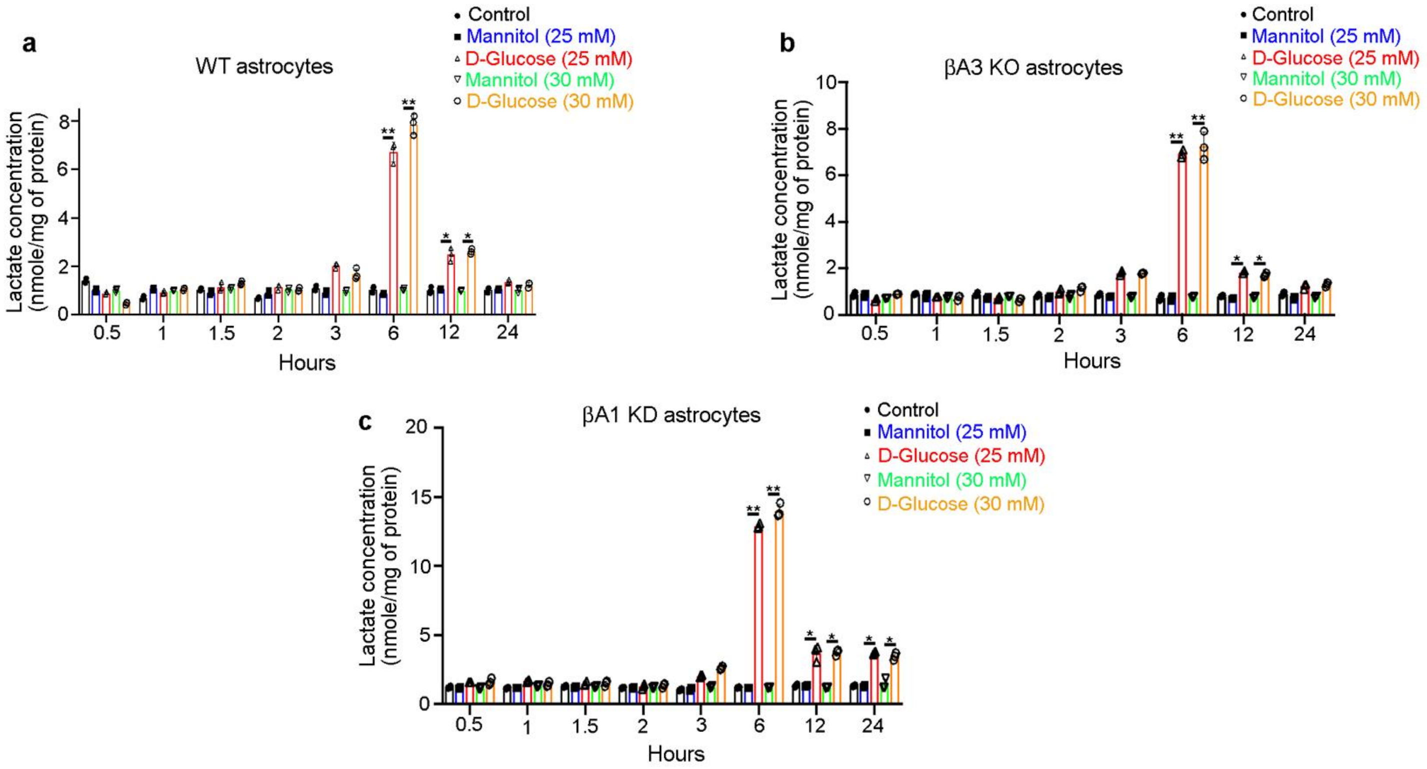

<u>Supplementary Figure 1</u>: **Glucose exposure to retinal astrocytes induce lactate production**. (**a**-**c**) WT, βA3 KO and βA1 KD astrocytes when exposed to 25 or 30 mM high glucose (HG) for 0.5, 1, 1.5, 2, 3, 6, 12 and 24 hours respectively, showed elevated lactate levels, compared to untreated cells after 6 hours of HG exposure which declined at later time points. Lactate levels in HG treated WT and βA3 KO astrocytes were similar to untreated cells at 24 hours, but it remained significantly higher in βA1 KD astrocytes even after 24 hours of HG exposure. n=4. *P<0.05, **P<0.01.

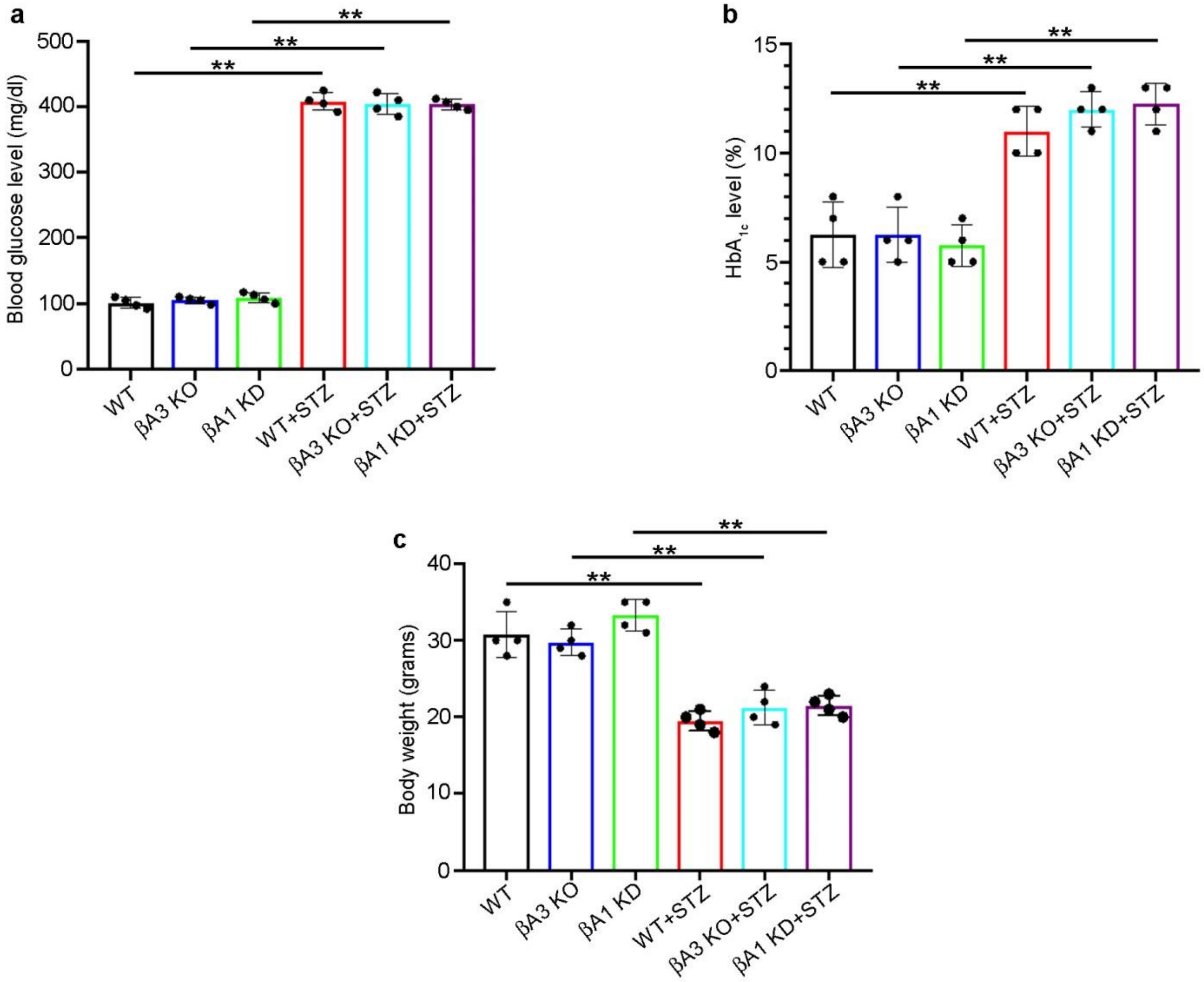

<u>Supplementary Figure 2</u>: **Generation of diabetic mice.** (**a**) Increase in blood glucose level and (**b**) HbA1c in diabetic (treated with a single intraperitoneal injection of streptozotocin; STZ at dose of 60 mg/kg body weight), 10 week old WT, βA3 KO and βA1 KD mice, compared to non-diabetic (intraperitoneally injected with vehicle; citrate buffer) controls. n=4. (**c**) Animals treated with STZ, did not show gain in body weight over the duration of the experiment, when compared to vehicle treated controls, further indicating onset of diabetes. n=4. **P*<0.05, ***P*<0.01.

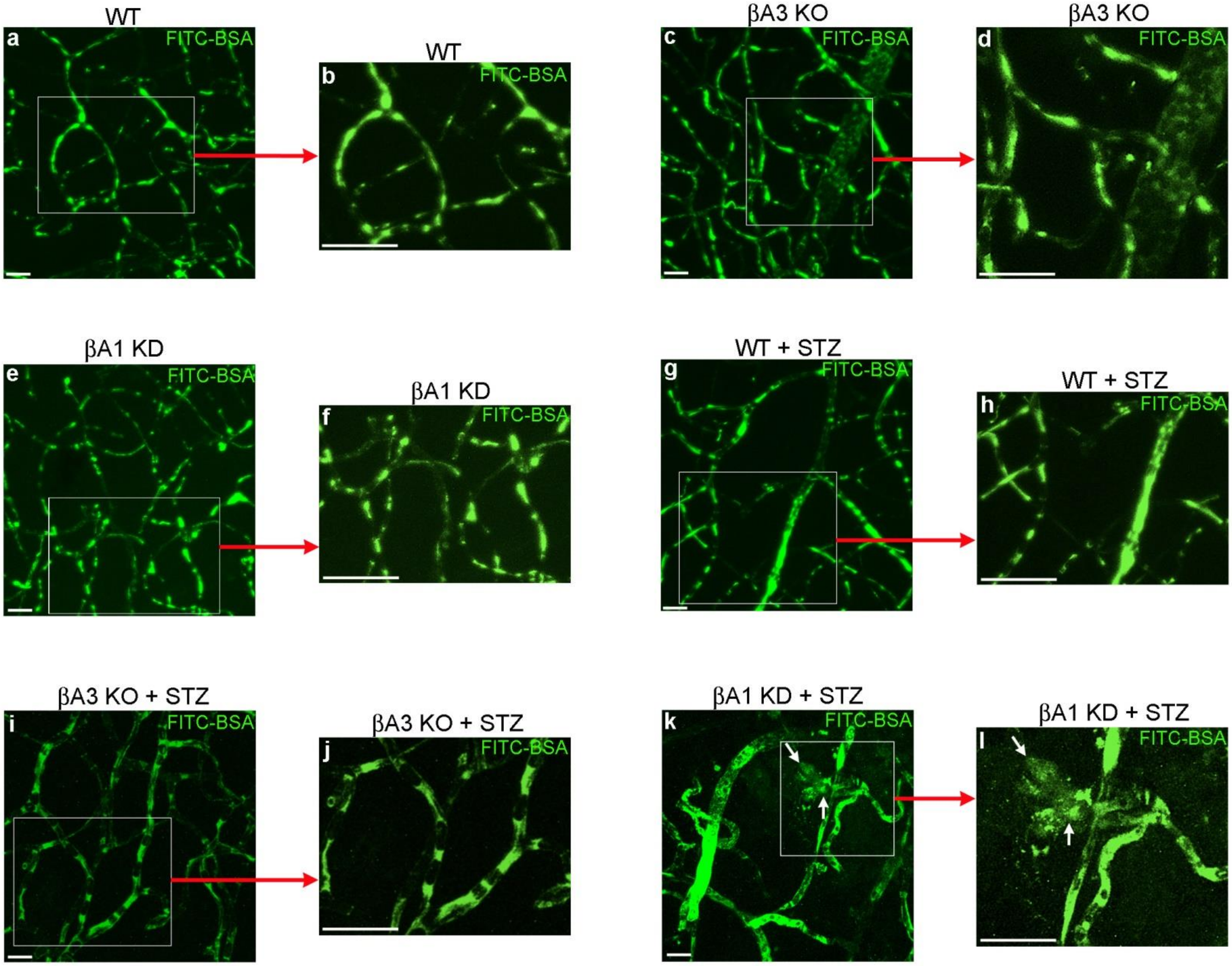

<u>Supplementary Figure 3</u>: **Assessment of vascular permeability**. Tail vein injection with FITC BSA in citrate buffer-treated (non-diabetic) or STZ-treated (diabetic) WT, βA3 KO and βA1 KD mice, followed by retinal flat mount preparation (please see methods) revealed noticeable vascular leakage in the diabetic βA1 KD mice (**k** and inset zoomed in **l**), as evident from the prevalence of the FITC-BSA fluorescence in the retinal tissue surrounding the blood vessel (arrows in **k** and **l**). Such changes in retinal vascular permeability were not observed in non-diabetic (**a**-**d**) or diabetic (**g**-**j**) WT and βA3 KO mice. n=4. Scale bar, 50 μM and Scale bar, 100 μM (inset).

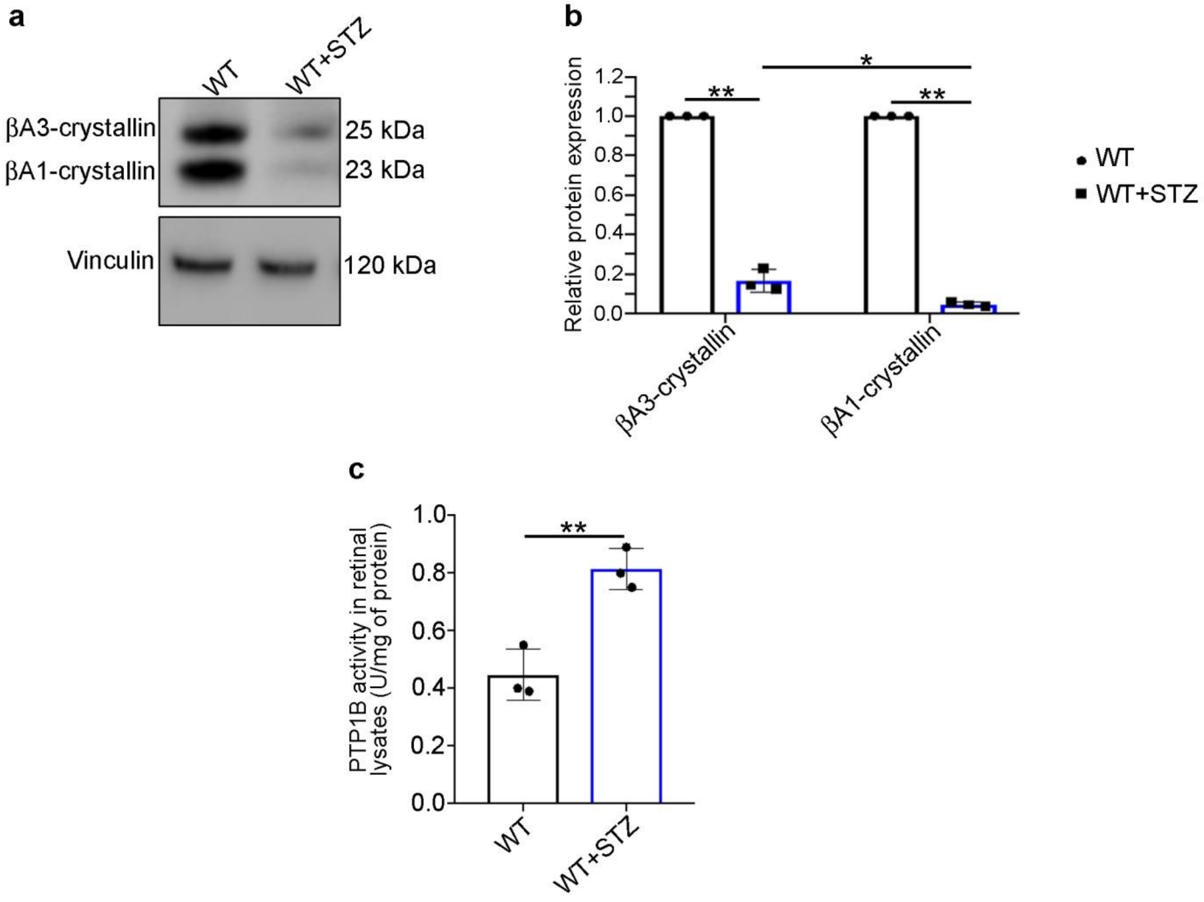

<u>**Supplementary Figure 4**</u>: **βA3/A1-crystallin expression and PTP1B activity in retinal lysates from diabetic and non-diabetic WT mice. (a)** Representative western blot and (**b**) densitometry graph showing decreased expression of βA3- and βA1-crystallin isoforms in retinal lysates from STZ-treated WT mice (10 months old), which were diabetic for 8 months, compared to non-diabetic (citrate buffer-treated) WT mice, where the expression of βA3- and βA1-crystallin isoforms showed a fold decrease of 84% and 96%, respectively, in the diabetic retinas relative to the non-diabetic. n=3. $**P<0.01$, $*P<0.05$. (**c**) Increase in PTP1B activity in the retina of STZ-treated (diabetic for 8 months) WT mice compared to non-diabetic (citrate buffer-treated) animals. n=3. $**P<0.01$.

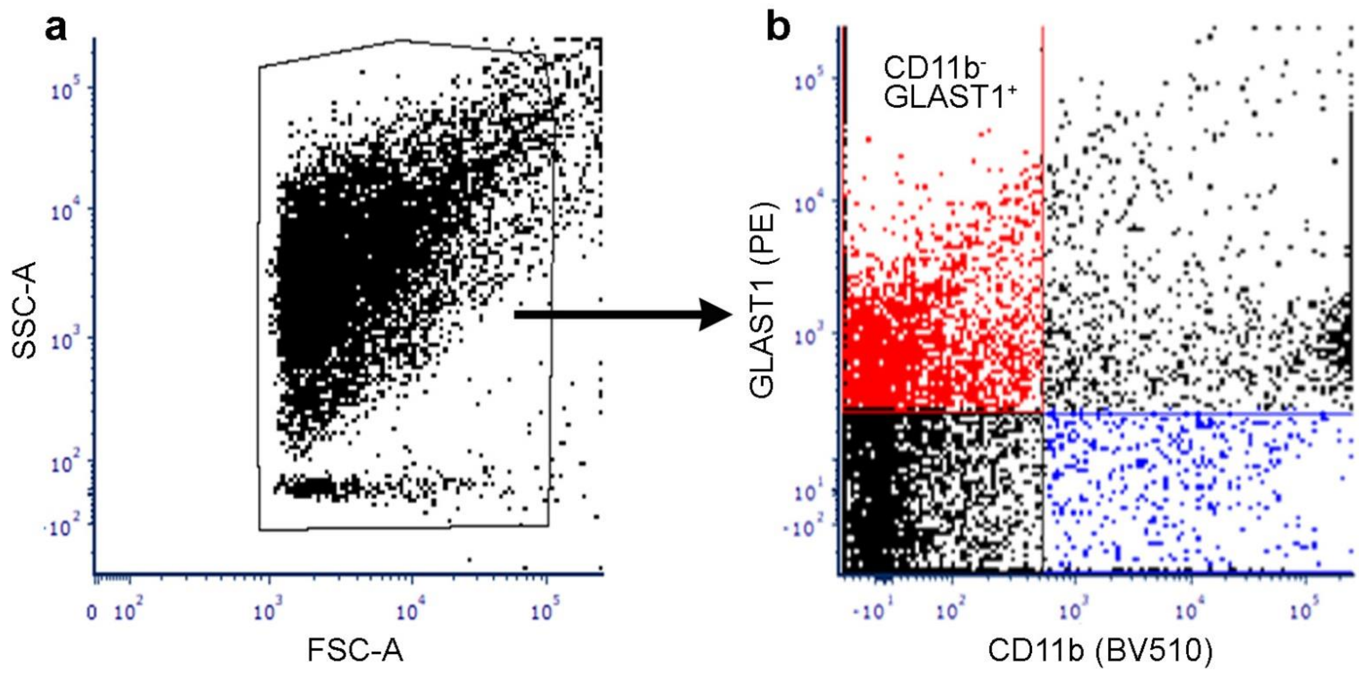

<u>Supplementary Figure 5</u>: Gating strategy for phenotyping the astrocyte population in vitreous humor. Representative images show gating strategy to identify astrocytes population in vitreous humor of study subjects. (**a**) The marked region Forward scatter area (FSC-A) versus Side Scatter area (SSC-A) represents the vitreous humor cell population that were analysed further for CD11b and GLAST1 staining. (**b**) Quadrant plot exhibits vitreous humor cell populations with varied GLAST1 (PE) and CD11b (BV510) staining. Upper left quadrant (red) indicates CD11b^{-}GLAST1^{+} cells and represent astrocytes. Upper right quadrant (large black) indicates CD11b^{+}GLAST1^{+} cells, lower left quadrant (small black) indicates CD11b^{-}GLAST1^{-} cells and lower right quadrant (blue) indicates CD11b^{+}GLAST1^{-} cells in vitreous humor. The proportion of astrocytes were calculated based on the numbers of CD11b^{-}GLAST1^{+} cells among the total number of cells (red) in the vitreous humor of each study subject.

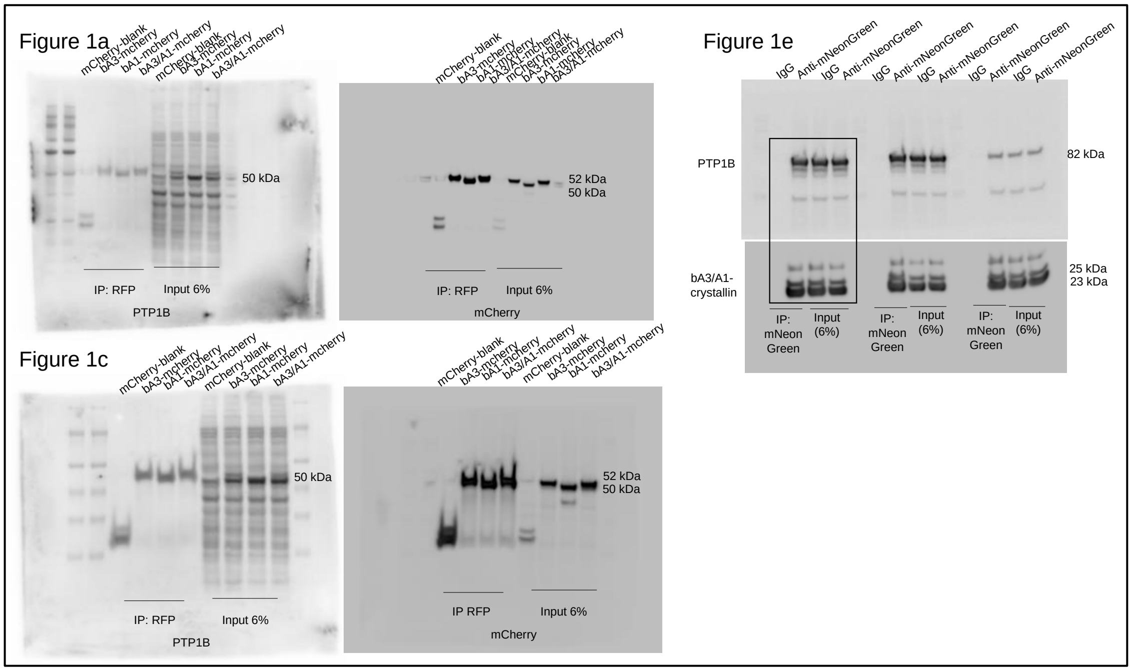
Figure 1a
mCherry-blank
bA3-mcherry
bA1-mcherry
bA3/A1-mcherry
50 kDa
IP: RFP
Input 6%
PTP1B
52 kDa
50 kDa
mCherry
Figure 1c
IP RFP
Figure 1e
IgG
Anti-mNeonGreen
PTP1B
82 kDa
bA3/A1-crystallin
25 kDa
23 kDa
IP: mNeon Green
Input (6%)

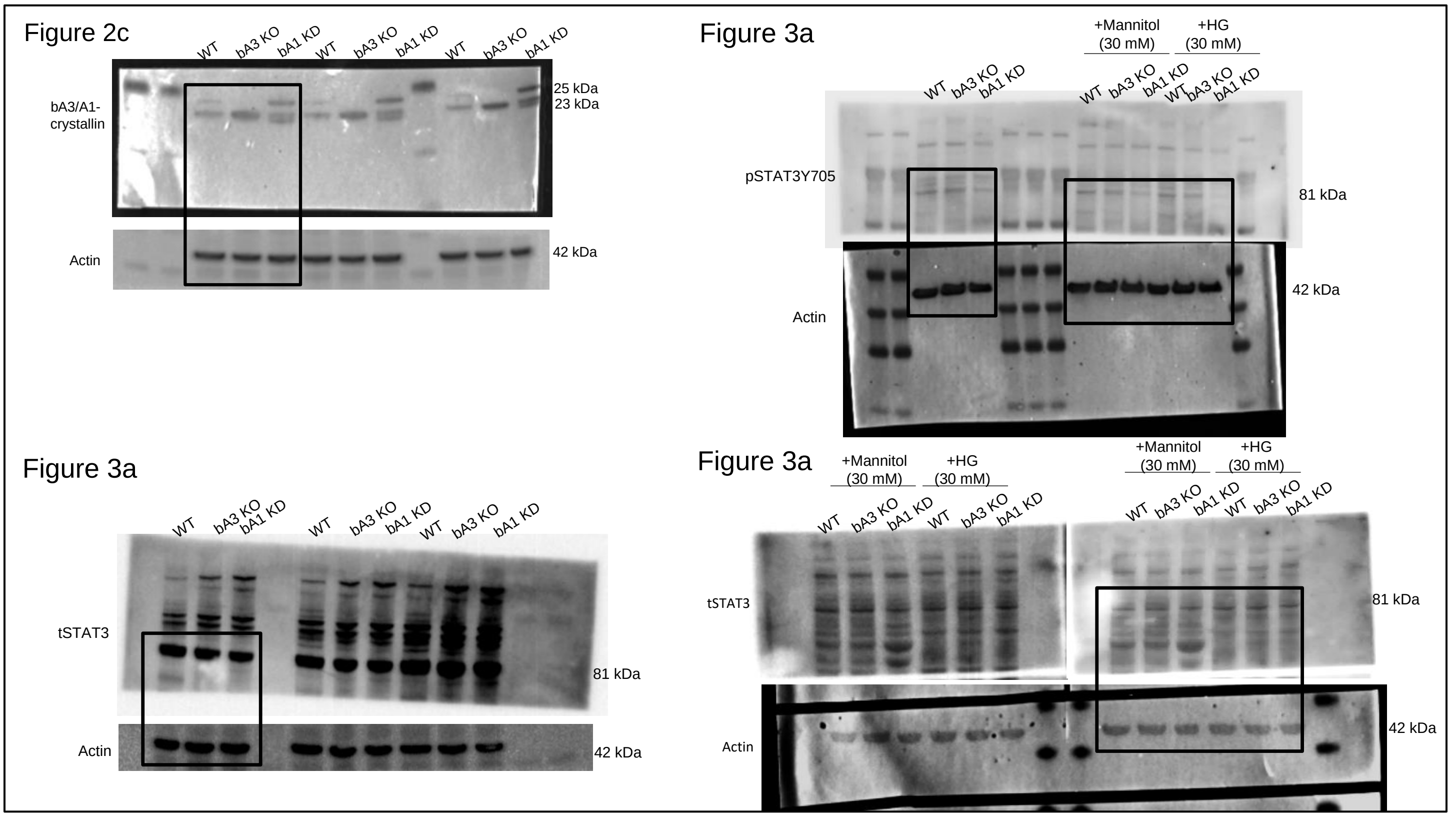
Figure 2c
WT
bA3 KO
bA1 KD
bA3/A1-crystallin
25 kDa
23 kDa
Actin
42 kDa
Figure 3a
+Mannitol (30 mM)
+HG (30 mM)
pSTAT3Y705
81 kDa
tSTAT3

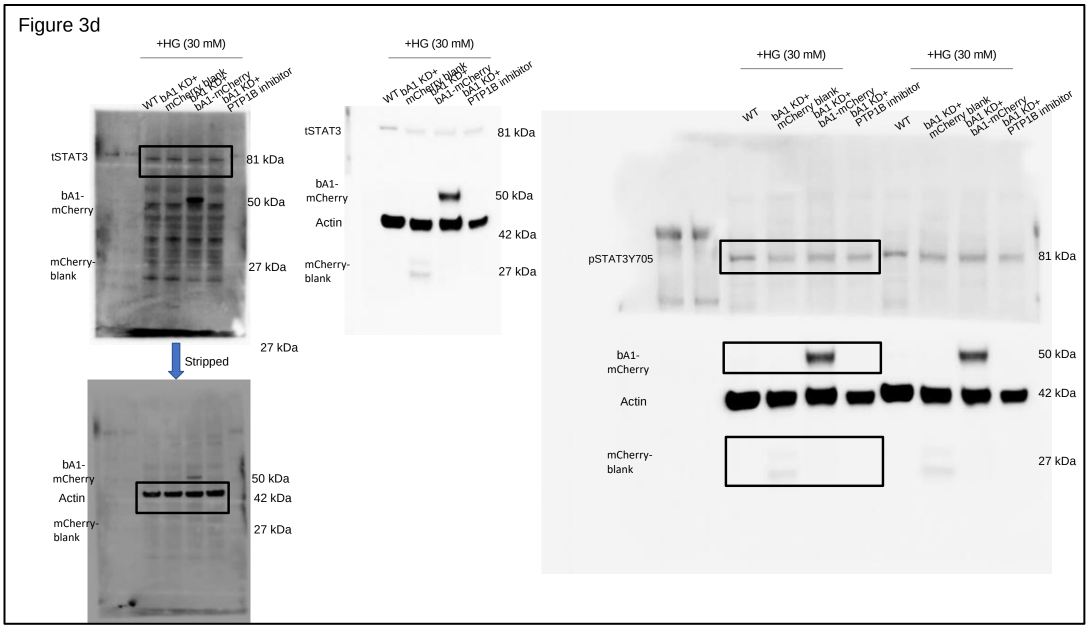
Figure 3d
+HG (30 mM)
WT
bA1 KD+ mCherry blank
bA1 KD+ bA1-mCherry
bA1 KD+ PTP1B inhibitor
tSTAT3
bA1-mCherry
mCherry-blank
Actin
Stripped
pSTAT3Y705
81 kDa
50 kDa
42 kDa
27 kDa

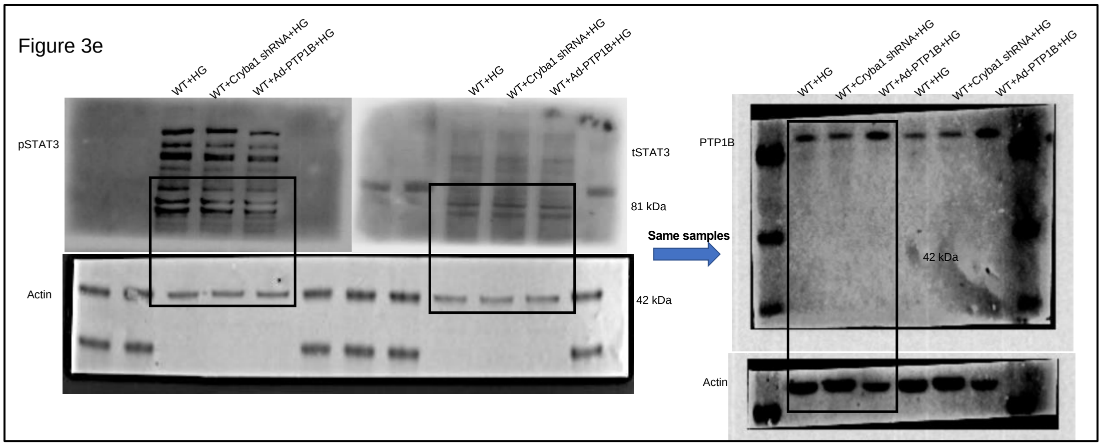
Figure 3e
WT+HG
WT+Cryba1 shRNA+HG
WT+Ad-PTP1B+HG
pSTAT3
tSTAT3
81 kDa
Same samples
PTP1B
42 kDa
Actin

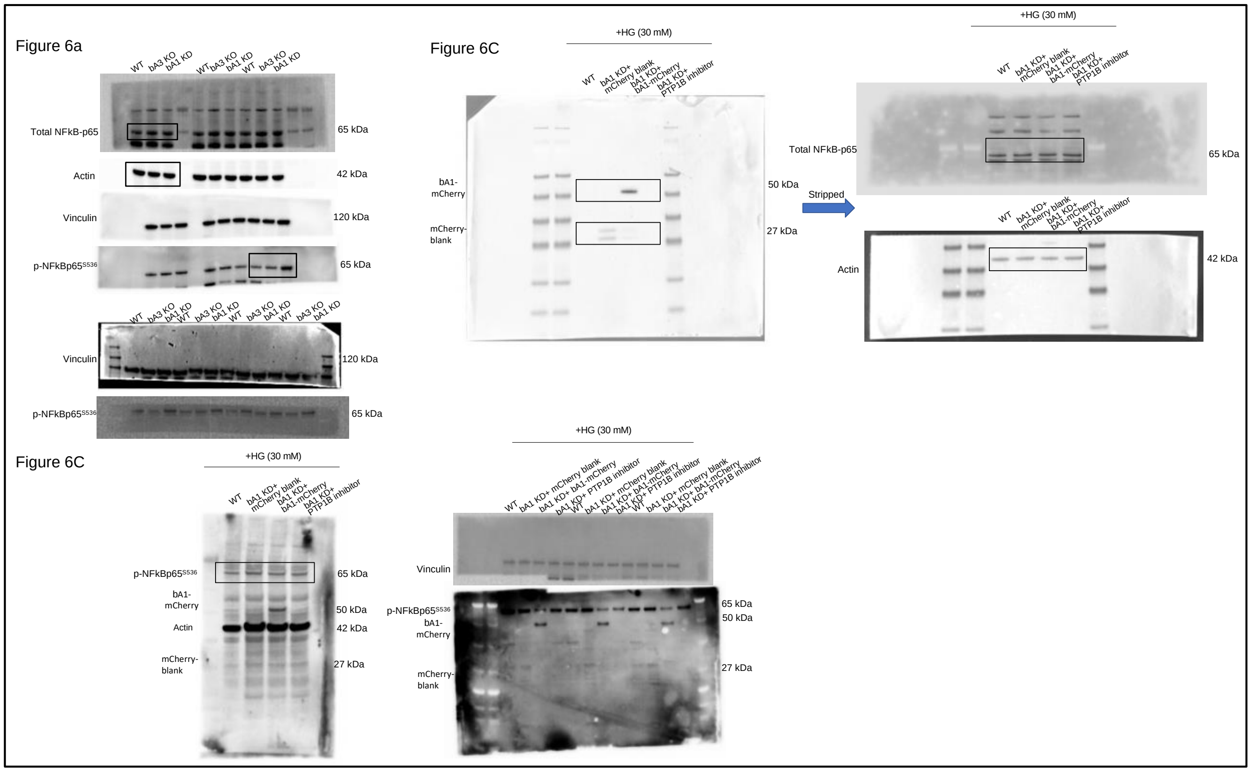
Figure 6a
WT
bA3 KO
bA1 KD
Total NFkB-p65
Actin
Vinculin
p-NFkBp65S536
65 kDa
42 kDa
120 kDa
Figure 6C
+HG (30 mM)
WT
bA1 KD+ mCherry blank
bA1 KD+ bA1-mCherry
bA1 KD+ PTP1B inhibitor
bA1-mCherry
mCherry-blank
50 kDa
27 kDa
Stripped

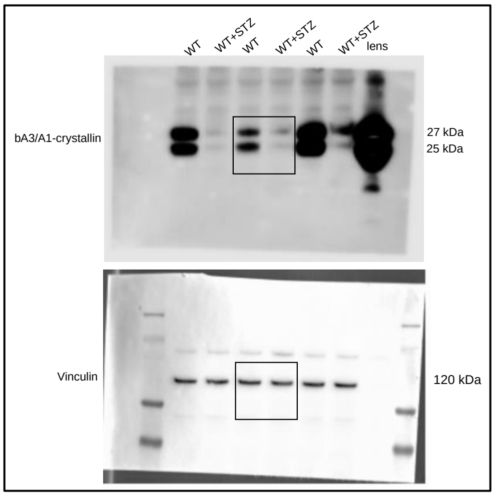

Supplementary Figure 6: The figure shows uncropped blots for all data for each figure panel represented in the manuscript. The bands incorporated in the manuscript are highlighted with black insets in the raw images.

Patient demographics for vitreous humor βA1-crystallin, PTP1B & soluble factors					
	**Control (n=14)**		**Proliferative diabetic retinopathy (n=23)**		
	Mean±SEM	Range	Mean±SEM	Range	P value
Age	56.92± 3.83	25-76 years	60.26 ± 1.49	49-74 years	0.6931
Gender (M/F)	7/7	NA	17/6	NA	NA

Supplementary Table 1: **Patient demographics for vitreous humor βA1-crystallin, PTP1B & soluble factors**

Correlation between soluble factors and PTP1B and βA1-crystallin				
Conc. (pg/μg)	**PTP1B**		**βA1-crystallin**	
	r	*P value*	*r*	*P value*
VEGF	0.607	0.0002	0.062	0.7172
IL-6	0.125	0.5110	-0.530	0.0013
IL-8	0.579	0.0002	-0.335	0.0424
MCP1	0.425	0.0191	-0.112	0.5266

Supplementary Table 2: **Relationship between PTP1B, βA1-crystallin and inflammation**. Table shows correlation coefficient values (*r*) between VEGF, IL-6, IL-8, MCP1 and PTP1B or βA1-crystallin levels in human vitreous humor samples (controls, n=14 and PDR patients n=23). *r* = Spearman Rank Correlation Coefficient.

Patient demographics for astrocyte measurement in vitreous humor					
	**Control (n=3)**		**Proliferative diabetic retinopathy (n=7)**		
	Mean±SEM	Range	Mean±SEM	Range	P value
Age	64.77 ± 2.91	60-70 years	55.43 ± 5.65	30-74 years	0.4167
Gender (M/F)	1/2	NA	5/2	NA	NA

Supplementary Table 3: Patient demographics for astrocytes measurement from vitreous humor
