# Supplementary material for: βA1-crystallin regulates glucose metabolism and mitochondrial function in mouse retinal astrocytes by modulating PTP1B activity
Source: Commun Biol. 2021 Feb 24;4:248. doi: 10.1038/s42003-021-01763-5 (PMC7904954; doi:10.1038/s42003-021-01763-5)
Supplement: Supplementary file 2 — Description of Supplementary Files [file 42003_2021_1763_MOESM2_ESM.pdf]

Description of Additional Supplementary Files

File name: Supplementary Movie 1
Description: The movie shows deconvoluted timeframe images of human iPSC-derived astrocytes transfected with (**a**) βA1-mCherry construct and (**b**) βA3-mCherry construct and imaged in a confocal microscope (please see Methods section). Scale bar, 10 μm.

File name: Supplementary Data 1
Description: Raw source data for all graphs and tables is represented in an Excel file and shows individual data points for each biological replicate.
