# Supplementary material for: βA1-crystallin regulates glucose metabolism and mitochondrial function in mouse retinal astrocytes by modulating PTP1B activity
Source: Commun Biol. 2021 Feb 24;4:248. doi: 10.1038/s42003-021-01763-5 (PMC7904954; doi:10.1038/s42003-021-01763-5)
Supplement: Supplementary file 5 — Reporting Summary [file 42003_2021_1763_MOESM5_ESM.pdf]

nature research

Corresponding author(s): Debasish Sinha

Last updated by author(s): Dec 16, 2020

Reporting Summary

Nature Research wishes to improve the reproducibility of the work that we publish. This form provides structure for consistency and transparency in reporting. For further information on Nature Research policies, see Authors & Referees and the Editorial Policy Checklist .

Statistics

For all statistical analyses, confirm that the following items are present in the figure legend, table legend, main text, or Methods section.

n/a	Confirmed	
☐	☒	The exact sample size (*n*) for each experimental group/condition, given as a discrete number and unit of measurement
☐	☒	A statement on whether measurements were taken from distinct samples or whether the same sample was measured repeatedly
☐	☒	The statistical test(s) used AND whether they are one- or two-sided<br>*Only common tests should be described solely by name; describe more complex techniques in the Methods section.*
☐	☒	A description of all covariates tested
☐	☒	A description of any assumptions or corrections, such as tests of normality and adjustment for multiple comparisons
☐	☒	A full description of the statistical parameters including central tendency (e.g. means) or other basic estimates (e.g. regression coefficient) AND variation (e.g. standard deviation) or associated estimates of uncertainty (e.g. confidence intervals)
☐	☒	For null hypothesis testing, the test statistic (e.g. *F*, *t*, *r*) with confidence intervals, effect sizes, degrees of freedom and *P* value noted<br>*Give P values as exact values whenever suitable.*
☒	☐	For Bayesian analysis, information on the choice of priors and Markov chain Monte Carlo settings
☒	☐	For hierarchical and complex designs, identification of the appropriate level for tests and full reporting of outcomes
☒	☐	Estimates of effect sizes (e.g. Cohen's *d*, Pearson's *r*), indicating how they were calculated

Our web collection on statistics for biologists contains articles on many of the points above.

Software and code

Policy information about availability of computer code

Data collection	BD FACSDiva software, Zeiss Zen Blue 2.1, HEX PROTEIN DOCKING version 6.3
Data analysis	Microsoft Excel, GraphPad Prism (versions 6 & 8), Cell Ranger (version 3.1.0), STAR Aligner (version 2.7), FlowJo software (v10.6.1), ImageJ, Seurat (version 3.1.0, 3.1.1)

For manuscripts utilizing custom algorithms or software that are central to the research but not yet described in published literature, software must be made available to editors/reviewers. We strongly encourage code deposition in a community repository (e.g. GitHub). See the Nature Research guidelines for submitting code & software for further information.

Data

Policy information about availability of data

All manuscripts must include a data availability statement. This statement should provide the following information, where applicable:
- Accession codes, unique identifiers, or web links for publicly available datasets
- A list of figures that have associated raw data
- A description of any restrictions on data availability

All data generated or analyzed during this study are included in this published article (and its Supplementary Data files).

Field-specific reporting

Please select the one below that is the best fit for your research. If you are not sure, read the appropriate sections before making your selection.

Life sciences study design

All studies must disclose on these points even when the disclosure is negative.

Sample size	Sample sizes was determined based on power calculation, showing a difference of 25% and up to 20% SD, alpha=0.05 and beta=0.2.
Data exclusions	No data were excluded, but some animals (<1 %) were excluded from the experiments due to technical challenges in the procedures such as animal death due to anesthesia complications.
Replication	At least three independent repeats were performed to reliably reproduce the results of the experiments.
Randomization	Animals from each genotype were allocated to each group randomly by separating in to different cages.
Blinding	To eliminate bias, core-facility technicians were blinded to mouse or rat genotype identity as well as the experimental groups.

Reporting for specific materials, systems and methods

We require information from authors about some types of materials, experimental systems and methods used in many studies. Here, indicate whether each material, system or method listed is relevant to your study. If you are not sure if a list item applies to your research, read the appropriate section before selecting a response.

Materials & experimental systems

n/a	Involved in the study	
☐	☒	Antibodies
☐	☒	Eukaryotic cell lines
☒	☐	Palaeontology
☐	☒	Animals and other organisms
☐	☒	Human research participants
☒	☐	Clinical data

Methods

n/a	Involved in the study	
☒	☐	ChIP-seq
☐	☒	Flow cytometry
☒	☐	MRI-based neuroimaging

Antibodies

Antibodies used	Primary antibodies: Phospho-Stat3 (Tyr705) (Thermo Fisher, USA; Cat# 44380G), p-NFkB p65 (S536) (Thermo Fisher, USA; Cat# MA515160), STAT3 (Thermo Fisher, USA; Cat# 10253-2-AP), beta Crystallin A3 (Abcam, USA; Cat# ab151722), IL-6 (Biorbyt, USA; Cat# orb6210), IL-1a (Biorbyt, USA; Cat# orb184287) and mNeonGreen (Chromotek, USA; Cat# 3216-100), Secondary antibodies: HRP anti-Rabbit IgG (KPL, USA; Cat# 074-1506), HRP anti-tagged anti-Mouse IgG (KPL, USA; Cat# 5220-0341), HRP anti-tagged Goat IgG (KPL, USA; Cat# 14-13-06). Primary antibodies were used at a dilution of 1:1000 whereas secondary antibodies were used at a dilution 1:2500 for western blotting.
Validation	The validation information for each antibody can be obtained from the catalog numbers mentioned in the methods section.

Eukaryotic cell lines

Policy information about cell lines

Cell line source(s)	Tempo's iAstro™: human iPSC-derived astrocytes are derived from integration-free induced pluripotent stem cell (iPSC) lines under a fully defined proprietary neural induction condition.
Authentication	Tempo's iAstro cells are polarized structures when plated as a monolayer in culture and express astrocyte markers like GFAP, EAAT1, Aquaporin family genes, and S100beta, and they exhibit calcium wave activities.
Mycoplasma contamination	We confirm that Tempo's iAstro cells tested negative for mycoplasma contamination.
Commonly misidentified lines (See ICLAC register)	N/A

Animals and other organisms

Policy information about studies involving animals; ARRIVE guidelines recommended for reporting animal research

Laboratory animals	Both male and female rats (Sprague Dawley) and mice (C57BL/6J background) were used in the study. No sex based difference was seen among the animals for any of the measured parameters in this study. Additional information regarding the genotype and age is provided in methods section.

Wild animals	N/A
Field-collected samples	N/A
Ethics oversight	All animal studies were conducted in accordance with the Guide for the Care and Use of Animals (National Academy Press) and were approved by the University of Pittsburgh Animal Care and Use Committee.

Note that full information on the approval of the study protocol must also be provided in the manuscript.

Human research participants

Policy information about [studies involving human research participants]

Population characteristics	Research participants who provided vitreous samples were from Indian decent.
Recruitment	Recruitment: Subjects were recruited for the study post informed written consent as per institutional and ethics board guidelines and as referred to Narayana Nethralaya Eye Hospital, Bangalore, India. The inclusion and exclusion criteria for the study are as follows: Inclusion Criteria: (i) Patients with proliferative diabetic retinopathy (PDR) confirmed with vascular proliferation at macula as diagnosed based on fundus imaging, OCT, and FFA. (ii) Subjects undergoing surgical intervention (as part of standard of care) that would require access into vitreous humor. Exclusion Criteria: (i) PDR patients with additional complications such as tractional retinal detachment and vitreous hemorrhage. Patients without any sign of vascular proliferation/abnormality in the retina but having other retinal conditions such as macular hole and floaters were scheduled for vitrectomy as a part of standard care and were considered as control.
Ethics oversight	All patient samples and related clinical information was collected post informed written consent as per institutional and ethics board guidelines and as referred to Narayana Nethralaya Eye Hospital, Bangalore, India.

Note that full information on the approval of the study protocol must also be provided in the manuscript.

Flow Cytometry

Plots

Confirm that:

- [x] The axis labels state the marker and fluorochrome used (e.g. CD4-FITC).
- [x] The axis scales are clearly visible. Include numbers along axes only for bottom left plot of group (a 'group' is an analysis of identical markers).
- [x] All plots are contour plots with outliers or pseudocolor plots.
- [x] A numerical value for number of cells or percentage (with statistics) is provided.

Methodology

Sample preparation	The detailed sample preparation for ail flow cytometry experiments are described in the methods section.
Instrument	BD FACSCantoII, BD Biosciences, USA and BD FACS Aria III, BD Biosciences, USA.
Software	BD FACSDiva software, FCAP array Version 3.0, FlowJo software (v10.6.1).
Cell population abundance	Sorting was not performed in this study.
Gating strategy	A detailed gating strategy is included in the methods section.

- [x] Tick this box to confirm that a figure exemplifying the gating strategy is provided in the Supplementary Information.
